# SlmA Antagonism of FtsZ Assembly Employs a Two-pronged Mechanism like MinCD

**DOI:** 10.1371/journal.pgen.1004460

**Published:** 2014-07-31

**Authors:** Shishen Du, Joe Lutkenhaus

**Affiliations:** Department of Microbiology, Molecular Genetics and Immunology, University of Kansas Medical Center, Kansas City, Kansas, United States of America; Max Planck Institute for Terrestrial Microbiology, Germany

## Abstract

Assembly of the Z-ring over unsegregated nucleoids is prevented by a process called nucleoid occlusion (NO), which in *Escherichia coli* is partially mediated by SlmA. SlmA is a Z ring antagonist that is spatially regulated and activated by binding to specific DNA sequences (SlmA binding sites, SBSs) more abundant in the origin proximal region of the chromosome. However, the mechanism by which SBS bound SlmA (activated form) antagonizes Z ring assembly is controversial. Here, we report the isolation and characterization of two FtsZ mutants, FtsZ-K190V and FtsZ-D86N that confer resistance to activated SlmA. In trying to understand the basis of resistance of these mutants, we confirmed that activated SlmA antagonizes FtsZ polymerization and determined these mutants were resistant, even though they still bind SlmA. Investigation of SlmA binding to FtsZ revealed activated SlmA binds to the conserved C-terminal tail of FtsZ and that the ability of activated SlmA to antagonize FtsZ assembly required the presence of the tail. Together, these results lead to a model in which SlmA binding to an SBS is activated to bind the tail of FtsZ resulting in further interaction with FtsZ leading to depolymerization of FtsZ polymers. This model is strikingly similar to the model for the inhibitory mechanism of the spatial inhibitor MinCD.

## Author Summary

Bacteria divide in the middle of the cell by spatially regulating the position of the Z ring, a cytoskeletal element required for cytokinesis. In the model organisms, *Escherichia coli* and *Bacillus subtilis*, two negative regulatory systems contribute to this spatial regulation. Both systems contain antagonists of FtsZ assembly that are localized in the cell. In this study we isolated FtsZ mutants resistant to SlmA, which is positioned within the cell by binding to sites asymmetrically distributed around the chromosome. We confirm that SlmA is activated by DNA binding to antagonize FtsZ polymerization *in vitro* and that the newly isolated mutants are resistant. We also show that SlmA binds to the very conserved tail of FtsZ and that this is required to antagonize FtsZ assembly even though the tail is not required for polymerization. Together, these results highlight the importance of the tail of FtsZ and lead to a model in which SlmA binding to the tail of FtsZ results in further interactions that break the filament. This mechanism is shared with the other spatial regulator and raises the possibility that it may be a common mechanism among spatial regulators of Z ring assembly.

## Introduction

The Z ring is a widely conserved cytoskeletal element required for prokaryotic cytokinesis [1]. It is assembled from polymers of FtsZ that are tethered to the membrane by interaction of the short conserved C-terminal tail of FtsZ with membrane anchoring proteins [2]–[4]. In the model organisms *E. coli* and *B. subtilis* the Z ring is restricted to midcell by the action of two negative regulatory systems, Min and nucleoid occlusion (NO) [3], [5]. The Min system antagonizes Z ring assembly away from midcell while NO prevents Z ring assembly over the nucleoid. In their absence, cells fail to divide due to FtsZ forming many spurious assemblies that are unable to mature into a functional Z ring [6], [7]. Both regulatory systems employ inhibitors of Z ring assembly that are dynamically positioned by interaction with structures within the cell. The products of the Min system interact with the membrane and the NO factors interact with the nucleoid [6]–[8].

The effector of the Min system is the FtsZ antagonist MinC, which is recruited to the membrane by MinD [9], [10]. The inhibitory MinC/MinD complex is positioned near the poles of the cell by MinE in *E. coli* and MinJ/DivIVA in *B. subtilis* [11]–[14]. MinC/MinD efficiently blocks Z ring formation by interacting with FtsZ filaments in two steps [9]. MinC/MinD first binds to the conserved tail of FtsZ through the C-terminal domain of MinC [15]. Binding to the tail of FtsZ within FtsZ filaments brings the N-terminal domain of MinC near the FtsZ filament, which is proposed to attack the filament, resulting in shortening of the filament [16].

The effectors of the NO system are SlmA in *E. coli* and Noc in *B. subtilis* [6], [7]. Both effectors are DNA binding proteins that are positioned within the cell by binding to specific sequences more prevalent in the origin proximal region of the chromosome [17]–[19]. SlmA is a member of the TetR family of repressors and has been shown to interact directly with FtsZ [7]. Noc is a member of the ParB family whose target is currently unknown [6]. These effectors are spatially regulated by the asymmetric distribution of their binding sites and the segregation of the replicating chromosome, which removes SlmA (Noc) from midcell [17]–[19].

Although it is established that binding to DNA activates SlmA to be an antagonist of Z ring assembly, there is disagreement over the mechanism. One study found that SlmA binding to an SBS activated SlmA to antagonize FtsZ polymerization [17]. Another study found that SlmA bound to an SBS as a dimer of dimers and that the dimers may spread from the SBS, however, antagonism of FtsZ polymerization was not observed [20]. It was proposed that the SlmA dimers bound to an SBS recruited FtsZ filaments and this prevented FtsZ filaments from coalescing into a Z ring [20]. A recent study identified residues on SlmA important for interaction with FtsZ [21], however, the SlmA binding site on FtsZ is not known. In this study we isolated FtsZ mutants that are resistant to SlmA. We confirmed that SlmA antagonizes FtsZ assembly and these mutants are resistant. We also found that SlmA binds the tail of FtsZ and this interaction is required for SlmA to antagonize FtsZ polymerization. Together, these results suggest a model for SlmA action that has similarities to the mechanism employed by MinC/MinD.

## Results

### Isolation of FtsZ mutants resistant to SlmA

FtsZ mutants resistant to SlmA were isolated by taking advantage of the observation that delocalization of SlmA from the nucleoid to SBS sites on a multicopy plasmid inhibits Z ring formation [17], [21]. The advantage of using this approach is that a 4 fold overexpression of SlmA blocks colony formation, whereas in the absence of a plasmid containing SBS sites a 40 fold increase in SlmA is required to block colony formation, which also blocks chromosome segregation [17], [21]. The coding region of *ftsZ* was subjected to PCR random mutagenesis and used to replace *ftsZ* in pBANG112, which produces close to (∼1.5×) the physiological level of FtsZ [15]. Three independent libraries were introduced into the strain DU11/pKD3C&pSD133 (*W3110 ftsZ^0^ recA::Tn10 slmA<>frt pKD3C [ftsZ^+^*] *& pSD133* [*P_tac_::slmA]*) and transformants selected at 42°C. Only a functional *ftsZ* on pBANG112 allows colony formation since pKD3C is temperature sensitive for replication. Transformants from each of the 3 libraries were separately pooled and transformed with plasmid p2SBSK, which contains two SBS sites. Transformants were selected in the presence of 20 µM IPTG since cells with wild type FtsZ are unable to form colonies at 10 µM IPTG and above 40 µM IPTG chromosome segregation is affected. Plasmids isolated from the survivors were retested to confirm their resistance and subjected to sequencing to identify *ftsZ* mutations.

**Figure 1:**
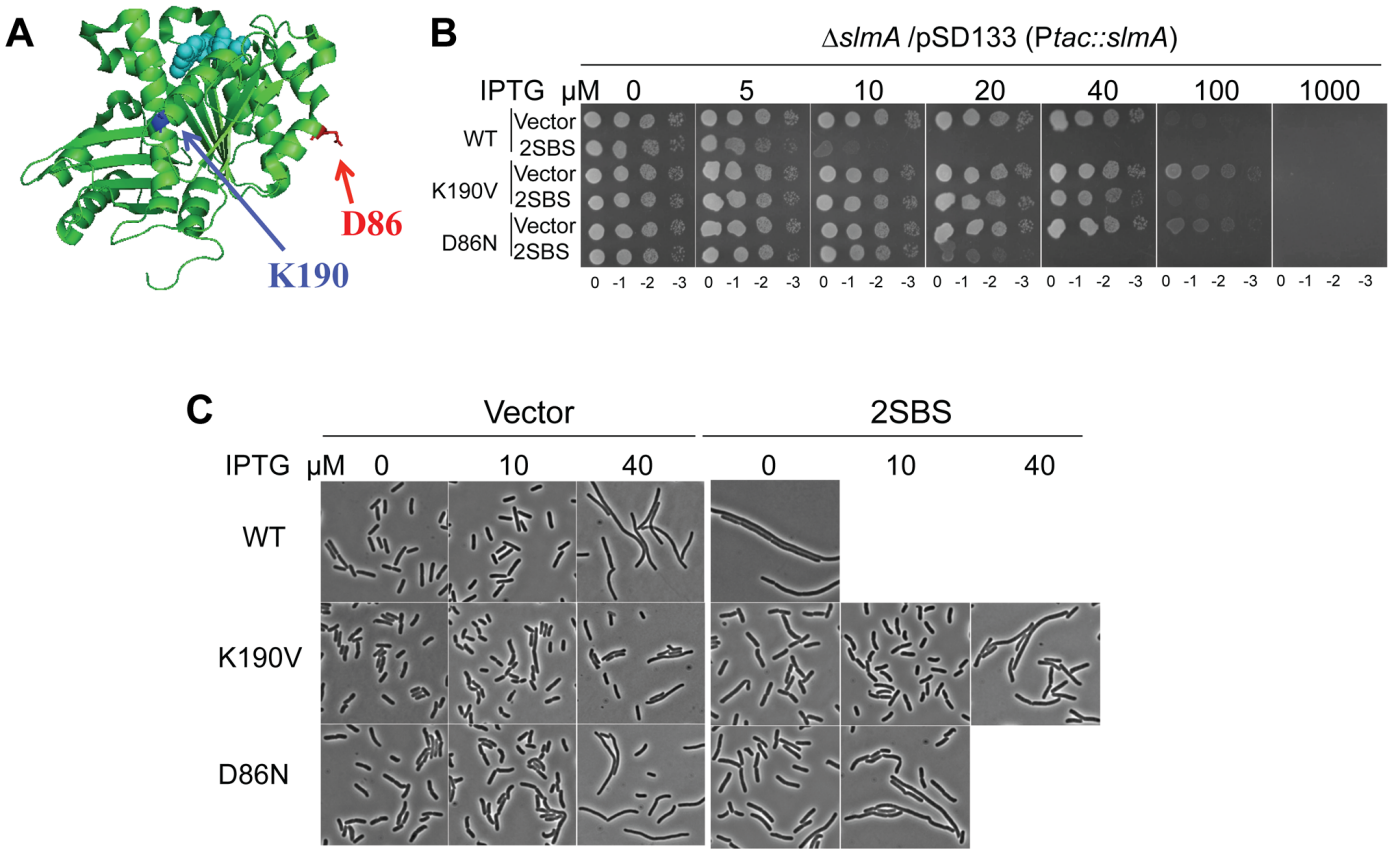
Mutations in two different regions of FtsZ confer resistance to de-localized SlmA. A) Locations of residues altered by the *ftsZ* mutations that showed resistance to delocalized SBS-SlmA. The structure of the FtsZ monomer from *Pseudomonas* (PDB# 1OFU) is shown and the residues corresponding to those mutated in *E. coli* are indicated. FtsZ-K190 (*A190 in Pseudomonas*) is in blue and FtsZ-D86 in red. B) Strains carrying *ftsZ-K190V* or *ftsZ-D86N* on the chromosome display significant resistance to SBS-SlmA. A spot test was carried out using the strains SD110 (*slmA::cat*), SD161 (*ftsZ-K190V slmA::cat*) and SD165 (*ftsZ-D86N slmA::cat*) containing the plasmids pSD133&p2SBSK or pSD133&pUC18K. C) Morphology of cells in B) grown in liquid culture with the indicated IPTG concentrations.

Sequence analysis revealed that most of these resistant mutants contained the same amino acid substitution (*ftsZ-K190I*) in helix H7 that connects the two sub-domains of FtsZ (Fig. 1A). Some of the *ftsZ-K190I* containing mutants also had other amino acid substitutions in *ftsZ*, but were not more resistant than the single mutant (Table S1). Another mutation, *ftsZ-D86N*, which alters a residue in helix H3 (Fig. 1A), was isolated twice, once as a double mutation *ftsZ-D86N&G95D* and once as a triple mutation *ftsZ-D86N&S246Y&M344I*. Subsequent analysis showed that the resistance was mainly due to *ftsZ-D86N* (Table S1). Despite screening 3 independent libraries, and verifying the quality of the mutagenesis by selecting and identifying mutations that confer resistance to MinCD (data not shown), these were the only mutations recovered.

Reintroduction of the above two mutations, *ftsZ-K190I* and *ftsZ-D86N*, into pBANG112 confirmed that they complemented the *ftsZ* depletion strain DU11/pKD3C at 42°C and conferred resistance to SlmA in the presence of p2SBSK (Fig. S1). Such complemented strains also grew at 37°C and 30°C, although both had a mild twisted-septal phenotype at or below 37°C (data not shown). Additional substitutions were introduced at these positions to see if mutants could be obtained that displayed resistance without altering the cell morphology. Changing *ftsZ-K190* to Ala, Leu, Glu, Asp, Trp and Val conferred various levels of resistance (Fig. S1). The *ftsZ-K190V* mutation produced a similar level of resistance as *ftsZ-K190I* and displayed a better morphology (Fig. S1). A positive charge at position 190 in FtsZ may be important for SlmA action since *ftsZ-K190R* was as sensitive to SlmA as the WT (Fig. S1 and Table S1). Changing D86 to Lys also reduced the sensitivity to SBS-SlmA, but the D86K substitution, as well as other substitutions at this position, either compromised the essential activity of FtsZ or had no effect on the resistance to SBS-SlmA (Fig. S1, Table S1). Since *ftsZ-K190V* and *ftsZ-D86N* displayed resistance to the delocalized SlmA with the least effect on cell morphology, they were chosen for further study.

### Characterization of FtsZ-K190V and FtsZ-D86N

To make sure the resistance phenotypes of *ftsZ-K190V* and *ftsZ-D86N* were not affected by the slightly higher expression level from the plasmid the mutations were introduced onto the chromosome at the *ftsZ* locus using the lambda RED recombineering system [22]. The resultant strains SD160 (*ftsZ-K190V*) and SD163 (*ftsZ-D86N*) grew as well as the wild type strain at all temperatures tested (Fig. S2B). The morphology of the cells, however, varied with temperature (Fig. S2A). At 42°C and 37°C, cells from both SD160 (*ftsZ-K190V*) and SD163 (*ftsZ-D86N*) looked similar to wild type, whereas at 30°C, a small fraction of SD160 (*ftsZ-K190V*) cells and a larger fraction of SD163 (*ftsZ-D86N*) cells displayed a twisted-septal morphology. Inactivation of *slmA* in these strains to create SD161 (*ftsZ-K190V slmA::cat*) and SD165 (*ftsZ-D86N slmA::cat*) did not result in any additional changes in morphology (Fig. 1C). Combining the *ftsZ-K190*V and *ftsZ-D86N* mutations (SD164 [*ftsZ-K190V ftsZ-D86N*]) resulted in cold sensitivity for growth and lysis at midcell so the combination was not studied further (Fig. S2).

The creation of the strains SD161 (*ftsZ-K190V slmA::cat*) and SD165 (*ftsZ-D86N slmA::cat*) allowed us to test the resistance of FtsZ-K190V and FtsZ-D86N to delocalized SBS-SlmA at the physiological level of FtsZ. As shown in Fig. 1B, the *ftsZ-WT* strain SD110 (*slmA::cat*) containing an SlmA expression plasmid (pSD133) along with a plasmid carrying two SBS sites (p2SBSK) failed to form colonies at or above 10 µM IPTG. However, the mutant strains harboring the same pair of plasmids survived at higher concentrations of IPTG, with SD161 (*ftsZ-K190V slmA::cat*) surviving at higher levels than SD165 (*ftsZ-D86N slmA::cat*). Microscopic examination of the *ftsZ-WT* strain (SD110 [*slmA::cat]*) revealed that exponentially growing cells were extremely filamentous even without IPTG induction whereas cells of strain SD161 (*ftsZ-K190V slmA::cat*) were not filamentous at 10 µM IPTG but became mildly elongated as the IPTG concentration was increased (Fig. 1C). The FtsZ-D86N mutant showed a similar pattern, but cells were more filamentous at equivalent IPTG concentrations (Fig. 1C). DAPI staining confirmed that there was no obvious DNA segregation problem at these IPTG concentrations (data not shown). Therefore, both *ftsZ-K190V* and *ftsZ-D86N* provide resistance to extra SBS bound SlmA, with *ftsZ-K190V* providing more resistance than *ftsZ-D86N*. The induction of SlmA in the absence of the SBS plasmid resulted in filamentation at 40 µM IPTG and inhibition of growth at 100 µM IPTG. The filamentation is due to FtsZ being recruited to the nucleoids [5] and FtsZ-K190V provides some resistance (Fig. 1C; vector panels). The inhibition of colony formation is due to inhibition of division and an effect on DNA segregation [17] and neither FtsZ mutant provides resistance to the segregation effect.

SlmA-T33A is a SlmA mutant unable to bind DNA and is therefore defective in nucleoid occlusion [17]. However, it disrupts Z ring formation when overproduced sufficiently *in vivo*, suggesting that the DNA-free form of SlmA can still interact with FtsZ and inhibit division [17]. Expression of SlmA-T33A from plasmid pSD128-T33A (this plasmid produces a higher level of SlmA than pSD133 due to a modification of the ribosome binding site) in SD110 (*slmA::cat*) prevented colony formation on plates with 40 µM IPTG or above (Fig. S3A). In contrast, strains SD161 (*ftsZ-K190V slmA::cat*) and SD165 (*ftsZ-D86N slmA::cat*) carrying pSD128-T33A (*slmA-T33*) survived at 1000 µM IPTG and 100 µM IPTG respectively. Microscopic analysis revealed that at 100 µM IPTG, the wild type strain was homogeneously filamentous whereas the FtsZ-K190V mutant was not and FtsZ-D86N was slightly elongated (Fig. S3B). Thus, these FtsZ mutants provide resistance to the unactivated SlmA (not DNA bound) as well as to activated SlmA.

### FtsZ-K190V and FtsZ-D86N are specifically resistant to SlmA

A reduction in the GTPase activity of FtsZ is a common nonspecific mechanism of resistance to inhibitors of FtsZ, such as SulA and MinC [23], [24]. If either *ftsZ-K190V* or *ftsZ-D86N* displayed resistance to these inhibitors, it would suggest that the GTPase activity of these mutants was compromised. However, neither *ftsZ-K190V* nor *ftsZ-D86N* displayed resistance to SulA (Fig. S4A). Also, microscopic examination of SD160 (*ftsZ-K190V*) and SD163 (*ftsZ-D86N*) in which MinCD was over expressed revealed that the cells were as filamentous as wild type cells expressing MinCD at each IPTG concentration examined (Fig. S4B). Consistent with this, neither SD160 (*ftsZ-K190V*) nor SD163 (*ftsZ-D86N*) produces minicells (Fig. S2). Thus, these alleles display no significant resistance to SulA or MinC indicating the resistance is specific to SlmA.

### Effect of FtsZ-K190V and FtsZ-D86N on nucleoid occlusion

Deletion of SlmA does not have a strong phenotype, but loss of SlmA is synthetic lethal with Δ*min* as the double mutant fails to assemble complete functional Z rings [7]. If the two FtsZ mutants are resistant to SlmA they should be synthetic lethal with *Δmin*. Although it is not completely understood, the synthetic lethality of Δ*min*Δ*slmA* is temperature sensitive as the double mutant grows at 42°C but not below 37°C [16]. Thus we created strains SD162 (*ftsZ-K190V min::kan*) and SD167 (*ftsZ-D86N min::kan*) at 42°C and then monitored their growth upon shift to 30°C. As reported, the Δ*min*Δ*slmA* double mutant DU5 (*min::kan slmA::cat*) could not form single colonies at 30°C on an LB plate, however, both SD162 (*ftsZ-K190V min::kan*) and SD167 (*ftsZ-D86N min::kan*) were able to grow at 30°C indicating they were not synthetic lethal with Δ*min* (Fig. S5A). Microscopic analysis of the cell morphology indicated that cells of SD162 (*ftsZ-K190V min::kan*) were much longer than the cells of the *min* deletion strain S4 (*min::kan*), while cells of SD167 (*ftsZ-D86N min::kan*) were similar to S4 (*min::kan*) (Fig. S5B). To quantify the difference between them, we measured the average cell lengths of all four strains grown at 42°C and after they were shifted to 30°C for two and a half hours. The *Δmin* strain only increased slightly in cell length after the shift, whereas the Δ*min*Δ*slmA* double mutant DU5 stopped dividing and the average cell length increased to 26.8 µm (Table S2). The average cell length of strain SD162 (*ftsZ-K190V min::kan*) increased from 6.8 µm to 16.7 µm indicating decreased division at 30°C. The cell length of SD167 (*ftsZ-D86N min::kan*) also increased, but it was similar to that of the *min* deletion strain S4 *min::kan*). These results indicate that the more resistant *ftsZ-K190V* is synthetic sick with Δ*min* while the less resistant *ftsZ-D86N* is not. Since some aspects of this test are not well understood, we sought an additional test.

**Figure 2:**
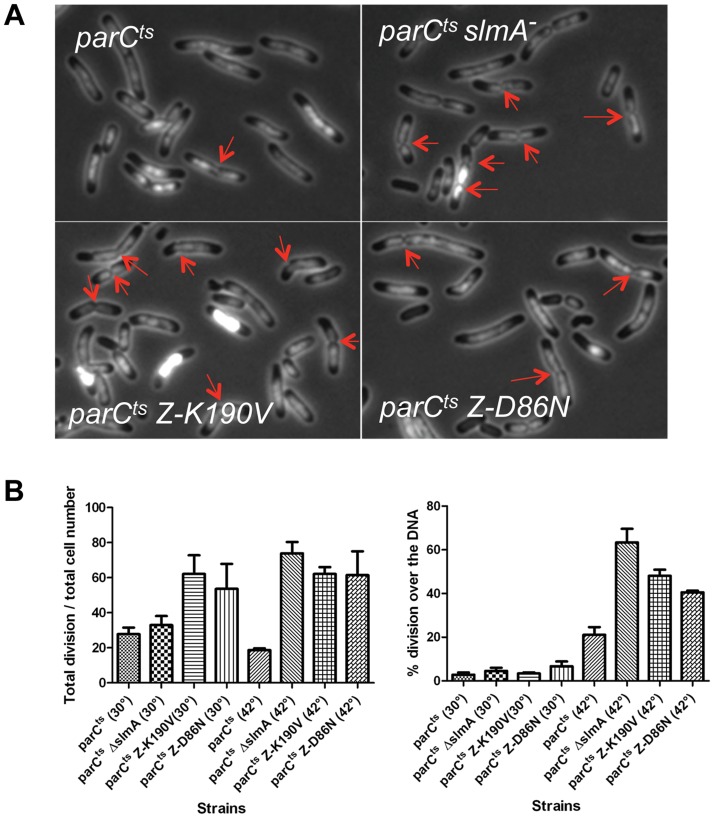
FtsZ-K190V and FtsZ-D86N are nonresponsive to SlmA mediated NO protection of unsegregated chromosomes. A) DAPI staining of cells shifted to 42°C for an hour. Exponentially growing cultures at 30°C were split into two parts, one of which was grown at 30°C and the other was shifted to 42°C. DAPI was added at a final concentration of 400 ng/ml 20 minutes before imaging. Red arrows indicate apparent constrictions over the DAPI staining. B) The number of septa in cells in (A) was quantified to calculate the percentage of cells with septa and the percentage of septa over unsegregated nucleoids. Data shown are the average of three experiments with more than 200 cells for each. Error bars indicate the standard deviation of the three experiments.

SlmA is required to prevent septation over an unreplicated nucleoid following DnaA depletion and is presumably responsible for the lack of septa forming over unsegregated nucleoids in a ParC^TS^ mutant at the nonpermissive temperature [7], [25]. To confirm this, SD139 (*parC^TS^*) and SD140 (*parC^TS^ slmA::cat*) were examined 1 hour after a shift to 42°C to inactivate ParC. DAPI was added to the culture shortly before microscopic examination so that septation over the DNA could be visualized. Cells containing WT SlmA (SD139 *parC* ^TS^) were mildly elongated and contained a single nucleoid mass (Fig. 2A). The percentage of cells with septa (17.6%; N = 1495) was about one half that of cells grown at 30°C (29.5%; N = 1278) and among the cells with septa, 21.7% (N = 263) were over the DNA (Fig. 2B). The percentage of cells with septa was much higher in the Δ*slmA* strain (SD140 *parC* ^TS^ *slmA::cat*) at the non-permissive temperature (74%; N = 955) and more than half of the septa were over the nucleoid (63.9%; N = 707). Therefore, SlmA mediated nucleoid occlusion is required to reduce the formation of septa over unsegregated chromosomes due to ParC deficiency.

To test if FtsZ-K190V and FtsZ-D86N were resistant to SlmA mediated nucleoid occlusion, strains SD170 (*parC^TS^ ftsZ-K190V*) and SD171 (*parC^TS^ ftsZ-D86N*) were generated by P1 mediated transduction. These strains were subjected to the same treatment as above and septa over the nucleoid were counted. Similar to SD140 (*parC^TS^ slmA::cat*), more than 60% of cells (62.5%; N = 1295) of the SD170 strain (*parC^TS^ ftsZ-K190V*) were observed with septa when shifted to non-permissive temperature, and about half of these septa (47.7%; N = 809) occurred over the nucleoid. Cells of SD171 (*parC^TS^ ftsZ-D86N*) displayed a similar percentage of cells with septa (62.5%; N = 1113) and the percentage of the septa over the nucleoid (40.7%; N = 698) was slightly lower, but still higher than the WT FtsZ control. Together, these results indicate that *ftsZ-K190V* and *ftsZ-D86N* allow septation over unsegregated nucleoids and are, therefore refractory to SlmA.

### SlmA antagonizes FtsZ assembly

**Figure 3:**
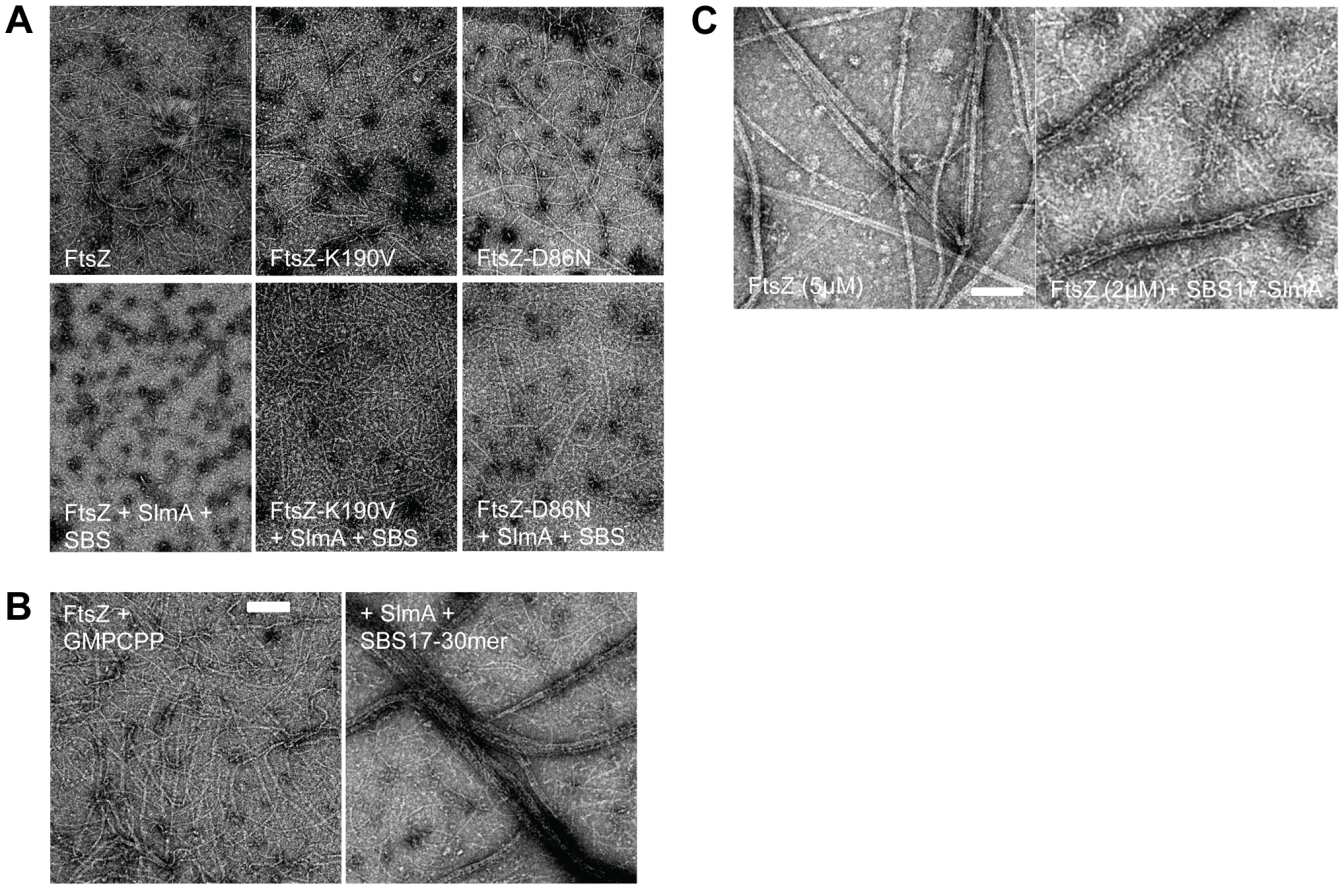
FtsZ mutants are resistant to SlmA antagonism of FtsZ polymerization. A) Polymerization of FtsZ with or without the addition of SlmA and SBS17-30mer. Reactions were performed in 50 µl volume containing FtsZ or one of the mutants (2 µM) and GTP (1 mM) with or without the addition of SlmA (2 µM) and SBS17-30mer DNA (2 µM) in FtsZ polymerization buffer (50 mM HEPES pH 8.0, 10 mM MgCl_2_, 200 mM KCl). Reactions were incubated at room temperature for 5 minutes before being spotted onto grids and negatively stained with 1% uranyl acetate for visualization by electron microscopy. B) Stable FtsZ filaments are bundled by SlmA/SBS. Reactions were carried out as in (A) except that GTP was replaced with GMPCPP. C) Comparison of FtsZ bundles and SBS-SlmA-FtsZ bundles obtained in the presence of GMPCPP. At 5 µM FtsZ assembles into bundles, which contain nicely aligned FtsZ proto-filaments. In contrast, the FtsZ bundles induced by SBS-SlmA have a rougher appearance and seen at lower FtsZ concentrations. Reactions were performed as in B).

The *in vivo* results demonstrate that FtsZ-K190V and FtsZ-D86N display resistance to the extra SBS/SlmA. We wanted to test their resistance *in vitro*, however, there is controversy in the literature about the effect SlmA has on FtsZ assembly. In one study [17] SlmA bound SBS antagonized FtsZ assembly whereas in another study [20] SlmA bound SBS had no effect on polymerization and it was suggested that SlmA/SBS affected higher order assembly of FtsZ filaments. To determine the effect of SlmA/SBS on FtsZ assembly we examined FtsZ polymerization by electron microscopy using purified 6×His-SlmA in the presence of SBS site 17 present on a 30mer (SBS17-30mer). The presence of 6×His-SlmA or the SBS17-30mer alone had no effect on FtsZ assembly, however, the presence of both dramatically decreased the amount and length of FtsZ polymers with the buffer routinely used in the laboratory (Fig. 3A; panels on left). This effect was also observed in both buffers used in the previous studies as well (Fig. S6). The polymers are mostly single-stranded under the conditions used due to the presence of 100–200 mM salt. To try and determine why different effects of SlmA/SBS were observed in the previous studies the effects of different DNAs were compared. Although all DNAs carried a similar SBS, the length of the flanking sequence varied. Reducing the length of the DNA from 30 to 20, 18 or 14 did not affect SlmA binding to the SBS but reduced the antagonistic effect on FtsZ assembly (Figs. S6B&S9C). With these shorter DNA fragments the abundance and length of polymers was still reduced, but not to the same extent as with the 30mer. It is not clear why decreasing the length of the flanking sequences decreased the activity of SlmA.

In the study that reported SlmA/SBS did not affect polymerization it was suggested that the polymers bound to the SlmA would clash and would be unable to propagate [20]. To test this we examined the effect of 6×His-SlmA/SBS17-30mer on stable FtsZ polymers formed in the presence of GMPCPP. Although FtsZ polymers have an increased tendency to bundle in the presence of GMPCPP (compared to GTP), the morphology was not influenced by the addition of SlmA or the SBS17-30mer alone. However, the addition of 6×His-SlmA and SBS17-30mer together led to a dramatic increase in bundling (Fig. 3B). This bundling was observed regardless of the length of the DNA containing the SBS (Fig. S6C). These bundles did not consist of simple alignment of FtsZ filaments, but had an altered appearance consistent with SBS bound SlmA bridging the FtsZ polymers (Fig. 3C). This result indicates that SlmA bound to DNA does not cause FtsZ filaments to clash such that their growth would be hindered.

### FtsZ-K190V and FtsZ-D86N display resistance to SBS-SlmA *in vitro*

Since SlmA bound to the SBS17-30mer caused a drastic reduction in the formation of polymers we proceeded to test its effect on the two FtsZ mutants. As shown in Fig. 3A, FtsZ-K190V (2 µM) assembled into mostly single-stranded filaments similar to WT FtsZ, whereas the filaments formed by FtsZ-D86N had an increased tendency to bundle. The enhanced bundling of FtsZ-D86N was more dramatic when the reactions were carried out at a higher concentration (5 µM) (data not shown). Polymers formed by two FtsZ mutants (FtsZ-K190V and FtsZ-D86N) were not detectably affected by the addition of either 6×His-SlmA (2 µM) or SBS17-30mer DNA (2 µM) alone (Fig. S7). Although the assembly of WT FtsZ was significantly reduced by the addition of 6×His-SlmA (2 µM) in the presence of SBS17-30mer DNA (2 µM), the assembly of FtsZ-K190V was only mildly affected; the filaments seemed to be somewhat shorter and not as smooth as FtsZ-K190V filaments without additions (Fig. 3A). Even though filaments formed by FtsZ-D86N were still readily detected upon addition of 6×His-SlmA (2 µM) in the presence of SBS17-30mer DNA (2 µM), the filaments and bundles were less and shorter compared to what was observed with FtsZ-K190V and 6×His-SlmA. These results indicate that these mutants display resistance to SlmA bound to an SBS *in vitro* with FtsZ-K190V displaying more resistance than FtsZ-D86N, consistent with its greater resistance to SlmA *in vivo*.

### FTsZ-K190V and FtsZ-D86N bind SlmA

There are at least two ways that mutations in *ftsZ* could confer resistance to SlmA: 1) substitution of important residues at the FtsZ-SlmA interaction interface or 2) lowering the GTPase activity of FtsZ. The latter seems unlikely since the mutants did not display resistance to SulA or MinC. Direct measurement of the GTPase activity revealed that both mutants displayed GTPase activity similar to wild type (Table S3). Therefore, we used several different tests to examine the interaction between the FtsZ mutants and SlmA. The two mutations did not affect the ability of FtsZ to associate with SlmA in the bacterial two-hybrid system as both mutants showed the same weak level of interaction with SlmA as wild type FtsZ (Fig. S8A).

**Figure 4:**
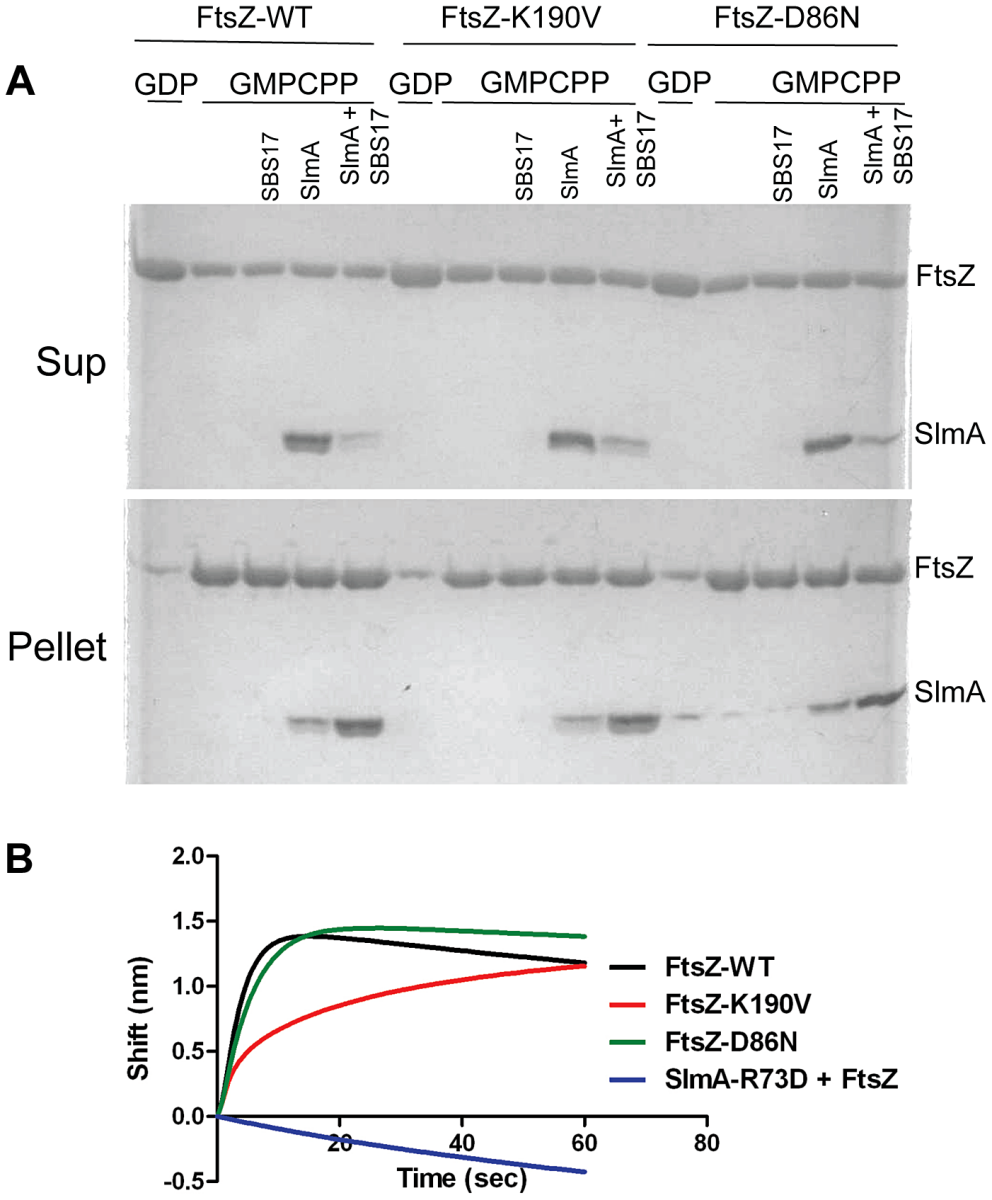
FtsZ-K190V and FtsZ-D86N bind SlmA. A) SBS17-30mer bound SlmA co-sediments with stable FtsZ polymers formed with GMPCPP. Polymerization assays were performed as in Fig. 3A except that the protein concentration was 5 µM and GTP was replaced by GMPCPP. After the reactions were incubated at room temperature for 5 minutes, FtsZ polymers were sedimented by ultracentrifugation. Proteins in the supernatant and pellet fractions were separated by SDS-PAGE. B) Biolayer interferometry assay to assess FtsZ binding to SlmA bound to DNA. Streptavidin biosensor tips loaded with biotin conjugated SBS17-30mer and SlmA were incubated with FtsZ or the FtsZ mutants (4 µM) and the association monitored.

Since the interaction in the two-hybrid system is weak several additional tests were done to examine the interaction. *Cho et. al* showed that SlmA bound to an SBS co-sedimented with stable FtsZ polymers (using an FtsZ mutant that was GTPase deficient) [17]. Stable polymers are also formed in the presence of the slowly hydrolysable analogue GMPCPP. As shown in Fig. 4A, the co-sedimentation of 6×His-SlmA (5 µM) with FtsZ-GMPCPP (5 µM) polymers was stimulated by the presence of SBS17-30mer DNA (2 µM). Control experiments showed that 6×His-SlmA-R73D (deficient in FtsZ interaction) did not co-sediment with FtsZ-GMPCPP in the presence of the SBS17-30mer (Fig. S8C) and 6×His-SlmA bound to SBS17-30mer did not sediment in the absence of FtsZ (Fig. S8B). Consistent with the bacterial two hybrid results, 6×His-SlmA bound to SBS17-30mer co-sedimented with FtsZ-K190V and FtsZ-D86N (Fig. 4A). Inspection of the reactions by electron microscopy supported these results. As expected, addition of 6×His-SlmA alone did not affect the morphology of the polymers, however, the addition of 6×His-SlmA and SBS17-30mer DNA caused dramatic bundling of the mutant FtsZ polymers, similar to what was observed with WT FtsZ (Fig. S8D).

In a third approach we examined the interaction of SlmA/SBS with unpolymerized FtsZ. In this assay 6×His-SlmA is bound to biotinylated SBS17-30mer immobilized on a streptavidin coated biosensor. The DNA binding mutant 6×His-SlmA-T33A, did not bind whereas the FtsZ interaction mutants, 6×His-SlmA-R73D and 6×His-SlmA-F65A bound to the DNA like WT SlmA (Fig. S9A) [17], [21]. The addition of FtsZ to the reaction containing 6×His-SlmA led to an increase in signal indicating binding, whereas the addition of FtsZ to the reaction containing 6×His-SlmA-R73D or 6×His-SlmA-F65A did not (Fig. S9B). Importantly, both FtsZ-K190V and FtsZ-D86N bound to SlmA to a similar extent as WT FtsZ (Fig. 4B), although FtsZ-K190V displayed slower binding kinetics. If the components in the assay were inverted (His-FtsZ or His-FtsZ-K190V immobilized on a Ni-NTA biosensor and SlmA/SBS17-30mer added), however, no difference in binding kinetics was observed (Fig. S9D). Thus, the resistance of FtsZ-K190V and FtsZ-D86N to SlmA does not appear to be due to a defect in the binding of activated SlmA to FtsZ.

Interaction was also examined by attaching SlmA directly to the biosensor. When 6×-His-SlmA was attached to a Ni-NTA biosensor FtsZ binding was only detected if SlmA was preincubated with the SBS17-30mer (data not shown). If 6×-His-FtsZ was bound to the biosensor no signal was observed with SlmA unless the concentration was increased to 50 µM (Fig. S9C). However, in the presence of SBS17-30mer, SlmA gave a strong signal at a much lower concentration (1 µM), consistent with previous finding that SBS DNA activates SlmA to bind to FtsZ [17]. The Kd for SlmA/SBS17-30mer binding to 6×-His-FtsZ was determined to be 0.21 µM, similar to that previously reported using another technique [19].

### FtsZ mutants resistant to MinC are not resistant to SlmA

Our data strongly supports the model where SBS associated SlmA prevents Z ring formation over the nucleoid by antagonizing FtsZ polymerization. However, the mechanism by which activated SlmA antagonizes FtsZ polymerization is not clear. Previous studies showed that activated SlmA stimulates the GTPase activity of FtsZ and requires the GTPase activity of FtsZ to disassemble FtsZ filaments [17]. Such a mechanism is very similar to that proposed for MinC in which the N-terminus of MinC interacts with the α10 helix of FtsZ resulting in a shortening of FtsZ polymers [16]. Interestingly, SAXS analysis of SlmA-FtsZ or SBS-SlmA-FtsZ complexes indicated that SlmA contacts FtsZ near helix 10 (Fig. S10A) [19], [20]. We therefore tested FtsZ mutants with substitutions in helix 10 that are resistant to the N-terminal domain of MinC [16]. A spot test showed that these mutants were actually slightly more sensitive to SlmA in the presence of extra SBSs (Fig. S10B). Therefore, even if the α10 helix is involved in SlmA binding, the way it interacts with SlmA must be different than with MinC. In addition, the fact that SlmA still co-sedimented with the FtsZ-GMPCPP polymers suggests that the α10 helix is unlikely to be the primary binding site for SlmA because it is partially occluded in the FtsZ-GMPCPP polymers.

### The conserved C-terminal tail of FtsZ is required for SlmA binding to FtsZ polymers and for susceptibility to SlmA

**Figure 5:**
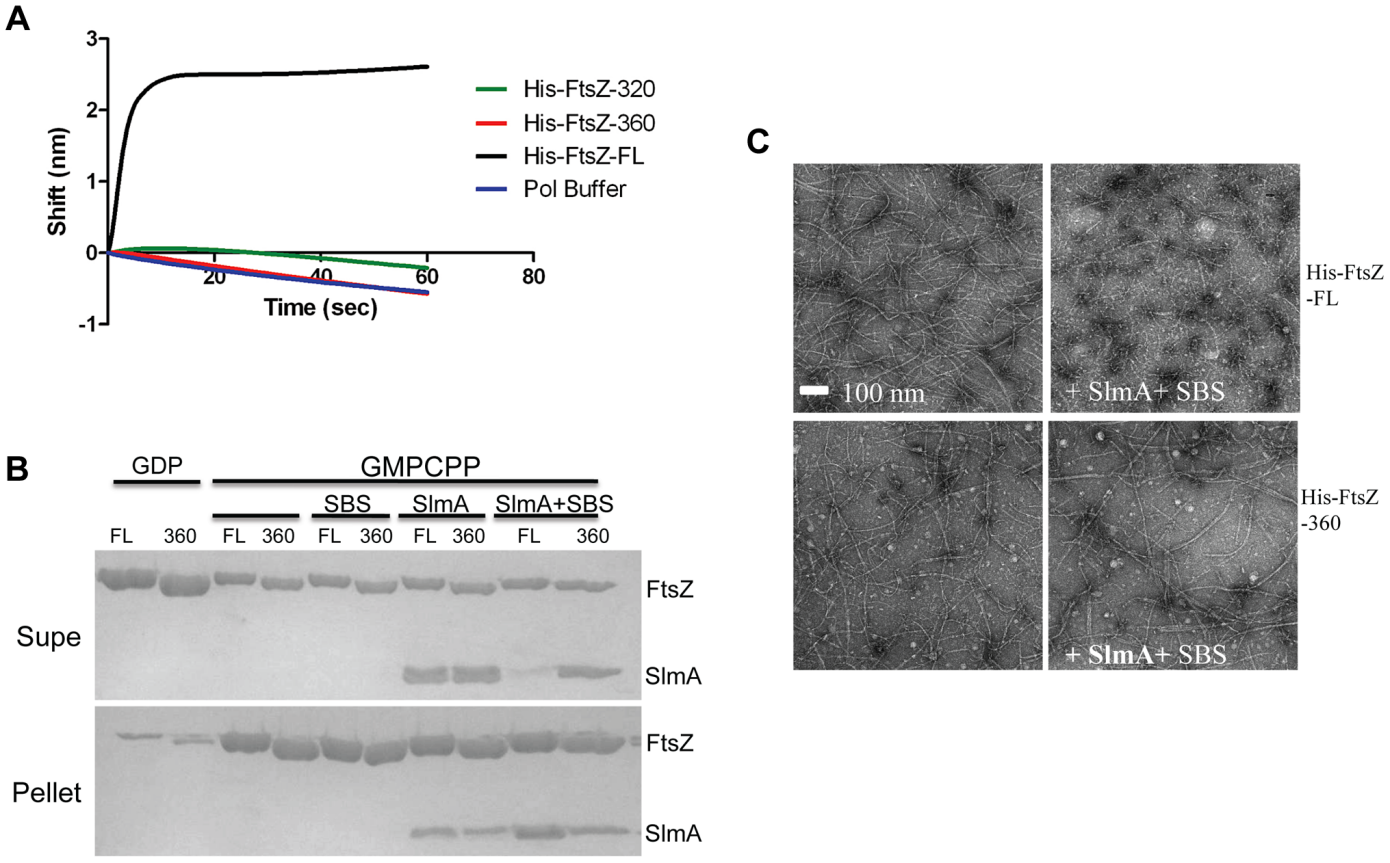
The conserved C-terminal tail of FtsZ is necessary for SlmA to bind to FtsZ and to antagonize FtsZ polymerization. A) Biosensor assay to monitor the binding of FtsZ C-terminal truncations to SlmA bound to biotinylated SBS17-30mer immobilized on a streptavidin biosensor. Reactions were performed as in Fig. 4B. B) SBS bound SlmA does not co-sediment with stable FtsZ_360_ polymers formed with GMPCPP. Reactions were performed as Fig. 4A. C) SBS-SlmA does not antagonize the assembly of C-terminally truncated FtsZ. His-FtsZ and His-FtsZ_360_ (2 µM) were incubated with GTP (1 mM) in the presence or absence of 6×His-SlmA and SBS17mer-30mer. Reactions were incubated at room temperature for 5 minutes and then samples were spotted onto carbon-coated grids for 1 minute, negatively stained with 1% uranyl acetate and visualized by electron microscopy.

**Figure 6:**
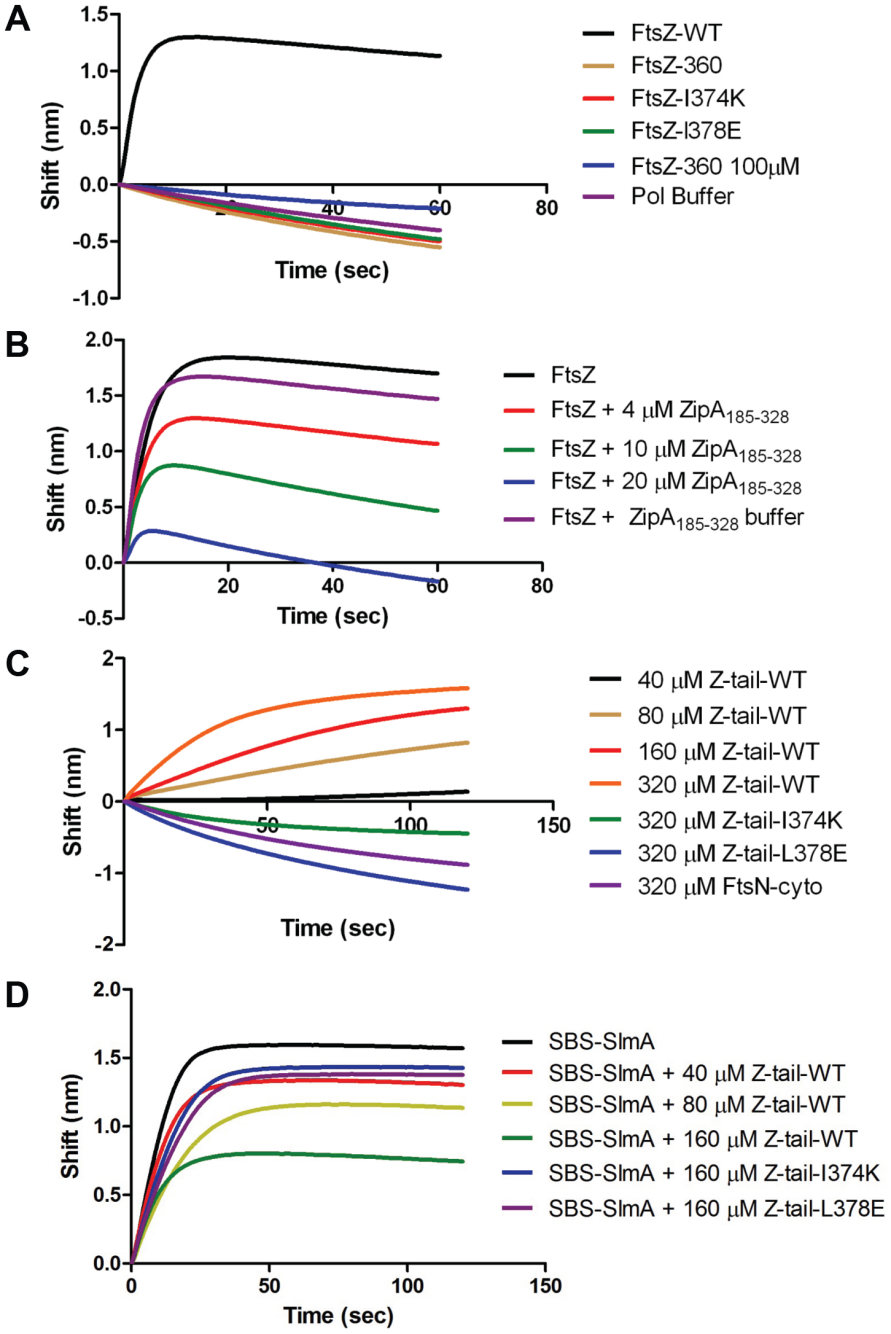
Further analysis of FtsZ-SlmA binding using biolayer interferometry. A) Single amino acid substitutions in the FtsZ tail abolish FtsZ-SlmA binding. The biotinylated SBS17-30mer was immobilized on a streptavidin biosensor and dipped into tubes containing 4 µM SlmA. Two minutes after incubation, the tips with SlmA bound to the biotinylated SBS17-30me were washed for 10 seconds and then moved to tubes containing FtsZ (4 µM) or its mutants and the association monitored. B) ZipA_185–328_ competes with SBS bound SlmA for the conserved C-terminal tail of FtsZ. Streptavidin biosensor tips were loaded with SlmA/SBS17-30mer complexes as before. The tips were washed for 10 seconds and then moved to tubes containing 4 µM FtsZ preincubated with various concentrations of ZipA_185–328_ and the association monitored. C) The FtsZ C-terminal tail peptide is sufficient for SBS-SlmA binding. Experiments were performed as in (A). Streptavidin biosensor tips were loaded with 50 nM biotinylated SBS17-30mer for 5 minutes followed by a 10 second wash. SBS17-30mer-SlmA complexes were generated by moving the SBS17-30mer coated tips to tubes containing 4 µM SlmA. The SlmA loaded tips were washed for 10 seconds and then moved to tubes containing FtsZ C-terminal tail peptide (Ztail-WT) or mutants to measure the association. A peptide corresponding to the cytoplasmic N-terminus of FtsN was also used as a nonspecific control. D) The FtsZ C terminal tail peptide competes with FtsZ for binding to SlmA-SBS. Streptavidin biosensor tips containing biotinylated SB17-30mer with bound SlmA were moved to tubes containing 4 µM FtsZ preincubated with various concentrations of the FtsZ C-terminal peptide and the association monitored.

The co-sedimentation of SlmA/SBS with stable FtsZ polymers indicates that activated SlmA must bind to the lateral surface of FtsZ polymers, the linker or the conserved C-terminal tail of FtsZ. A previous study reported that the conserved C-terminal tail of FtsZ was not required for SlmA binding [19]. To confirm this, we tested 6×His-FtsZ_320_, which lacks the linker and the conserved C-terminal tail, to see if it would bind to SlmA using the biosensor assay. Surprisingly, 6×His-FtsZ_320_ did not bind to SlmA/SBS17-30mer immobilized on the biosensor (Fig. 5A) suggesting that either the linker region or the conserved C-terminal tail is necessary for SlmA binding. We next tested 6×His-FtsZ_360_, which also did not bind, demonstrating the conserved C-terminal tail, and not the linker, is required for SlmA binding (Fig. 5A). To ensure this lack of binding was not due to the 6×His tag, we tested if FtsZ_360_ bound to the SlmA/SBS17-30mer. Consistent with the other results, FtsZ_360_ exhibited no binding signal with SlmA/SBS17-30mer, while 6×His-FtsZ and FtsZ both bound to SlmA/SBS17-30mer preloaded on the biosensor (Fig. 5A and Fig. 6A). Thus, the conserved C-terminal tail of FtsZ is required for SlmA to bind to FtsZ.

To test if the conserved tail of FtsZ is required for interaction between SlmA and FtsZ polymers, we tested whether SlmA bound to a SBS17-30mer co-sedimented with stable 6×His-FtsZ_360_ polymers assembled with GMPCPP. As shown in Fig. 5B, SBS17-30mer DNA stimulated SlmA to codsediment with FtsZ but not with FtsZ_360_ stable polymers. We also visualized the effect of SlmA bound to SBS17-30mer DNA on the morphology of the stable polymers. The dramatic bundling of the stable 6×His-FtsZ polymers induced by SlmA/SBS-30mer, was not observed with stable 6×His-FtsZ_360_ polymers (Fig. S11). Therefore, the conserved C-terminal tail of FtsZ is also necessary for SlmA to interact with polymerized FtsZ.

We next checked whether the conserved C-terminal tail of FtsZ was required for SBS-SlmA induced disassembly of FtsZ polymers. As shown in Fig. 5C, 6×His-FtsZ_360_, like 6×His-FtsZ, assembled into single and double stranded filaments in the absence of SlmA. The addition of SlmA along with SBS17-30mer DNA dramatically reduced the number and length of 6×His-FtsZ polymers but had no effect on 6×His-FtsZ_360_ polymers, indicating that the conserved C-terminal tail of FtsZ is required for disassembly of FtsZ polymers by SlmA bound to SBS17-30mer. This appears counterintuitive since the tail of FtsZ is not required for polymerization.

### Single substitutions in the conserved C-terminal tail of FtsZ abolish the FtsZ-SlmA interaction

The C-terminal tail of FtsZ (DYLDIPAFLRKQAD_383_) is widely conserved in bacteria and many proteins involved in cell division have been reported to bind to the tail of FtsZ. In *E. coli*, there is evidence that the tail of FtsZ interacts with five different proteins, ZipA, FtsA, MinC, ZapD and ClpX [2], [15], [26]–[28]. FtsZ residues I374 and L378 are critical for the interaction with ZipA, FtsA and MinC [15], [26]. Depending upon the amino acid substitutions at these two positions resistance to MinC-MinD or loss of interaction with ZipA and FtsA have been reported [16], [26]. As the tail of FtsZ is required for FtsZ-SlmA interaction, it is highly likely that substitution of these two residues would also disrupt the FtsZ-SlmA interaction. Therefore, we generated FtsZ-I374K and FtsZ-L378E and tested their interaction with SlmA in the biosensor assay. As shown in Fig. 6A these mutants behaved like FtsZ_360_ as no binding signal was observed with SlmA bound to the SBS17-30mer immobilized on the biosensor. Thus, I374 and L378 are also important for the FtsZ-SlmA interaction.

### ZipA competes with SlmA for the conserved C-terminal tail of FtsZ

Previous studies revealed that ZipA and FtsA bind to the conserved C-terminal tail of FtsZ, although there are subtle differences in the sequence specificity [26]. Nonetheless, these proteins should compete with SlmA for binding to FtsZ. We therefore tested the C-terminal domain of ZipA, ZipA_185–328_, which binds to FtsZ with high affinity [29], [30]. As shown in Fig. 6B preincubation of FtsZ with ZipA_185–328_ prevented FtsZ binding to SlmA/SBS17-30mer in a concentration dependent manner. At a 1∶1 ratio of FtsZ to ZipA_185–328_ the binding signal for FtsZ with SlmA/SBS17-30mer decreased about 30% and at a 1∶5 ratio the binding signal was almost completely eliminated. These data demonstrate that SlmA competes with ZipA for the conserved tail of FtsZ indicating that the binding sites for these two proteins overlap.

### The FtsZ tail peptide is sufficient for SlmA binding and blocks SBS-SlmA binding to FtsZ

Previous studies have shown that a peptide corresponding to the C-terminal tail of FtsZ binds specifically to ZipA and FtsA [30], [31]. Therefore, we tested a synthetic 14 amino acid peptide corresponding to the conserved C-terminal tail of FtsZ (Ztail-WT, DYLDIPAFLRKQAD) for binding to SlmA bound to SBS17-30mer. As shown in Fig. 6C, this peptide bound to SBS17-30mer-SlmA in a concentration dependent manner. Analysis of the binding curves revealed that the Kd for the peptide binding to SBS17-30mer-SlmA was 81±10 µM, dramatically lower than for full length FtsZ (0.21 µM; Table S4). However, this is not unusual because the FtsZ tail peptide also displays low binding affinity for ZipA (20 µM) and FtsA (50 µM) [30], [31]. Consistent with the results obtained above with the full length FtsZ tail mutants, the mutant peptides, Ztail-I374K and Ztail-L378E, did not bind to SlmA/SBS17-30mer even at the highest concentration tested (320 µM) (Fig. 6C). Furthermore, a peptide corresponding to the cytoplasmic domain of FtsN failed to bind SlmA. To further examine the FtsZ-SlmA interaction we looked at the ability of the Ztail-WT peptide to compete with FtsZ for binding to SlmA-SBS. Addition of the Ztail-WT peptide, but not mutant peptides (Z-tail-I374K and Z-tail-L378E), led to a concentration dependent decrease in the binding of full length FtsZ (Fig. 6D). Together, these results indicate that the binding of the Ztail-WT peptide to SlmA is specific.

### Evidence that the SlmA FtsZ-C-terminal tail interaction is important *in vivo*

**Figure 7:**
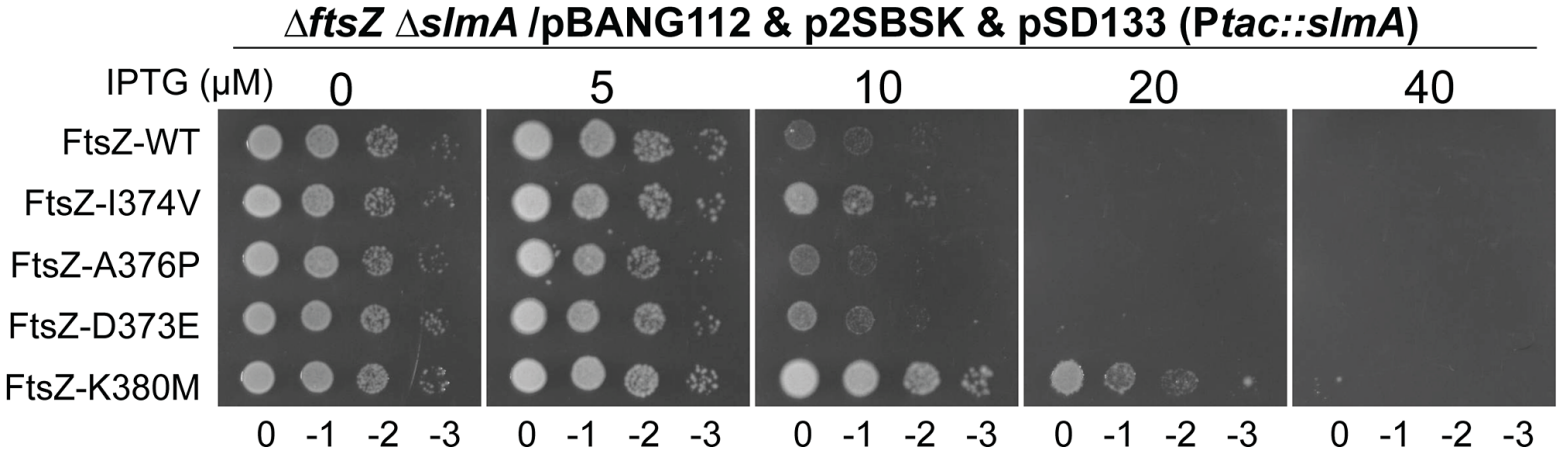
FtsZ tail mutants resistant to MinC/MinD display differential sensitivity to de-localized SlmA. Plasmids pSD133 (P*tac::slmA*) and p2SBSK (pUC18 with 2SBS sites) were introduced into the *ftsZ^−^* strain DU11 (*ftsZ^0^ slmAfrt<>recA::Tn10*) complemented with pBANG112 or its derivatives containing different *ftsZ* alleles. One colony of each resultant strain was resuspended in 1 ml LB medium, serially diluted by 10 and 3 µl from each dilution was spotted on LB plates containing various concentrations of IPTG and supplemented with antibiotics. The plates were incubated at 30°C for 30 hours before being photographed.

Some FtsZ tail mutants are insensitive to the division inhibitory activity of MinC^C^-MinD [15]. These FtsZ mutants, including Fts-D373E, I374V, A376P (unpublished data), L378V and K380M, likely eliminate or reduce the interaction between FtsZ and MinC^C^ but retain interaction with ZipA and FtsA (they can replace WT FtsZ) [15]. Each of these FtsZ mutants complemented DU11/pKD3C at 42°C when expressed from pBANG112, however, DU11/pBANG112-L378V grew poorly and was not studied further. The well characterized FtsZ-I374V mutant was still sensitive to SlmA, indicating that although both SlmA and MinC bind to the conserved C-terminal tail of FtsZ, they must interact with the tail differently (Fig. 7). Most of the other mutants were also sensitive, however, FtsZ-K380M displayed a weak resistance to SlmA. The resistance of FtsZ-K380M to SlmA provides evidence that the interaction of SlmA with the conserved C-terminal tail of FtsZ is important *in vivo*.

## Discussion

**Figure 8:**
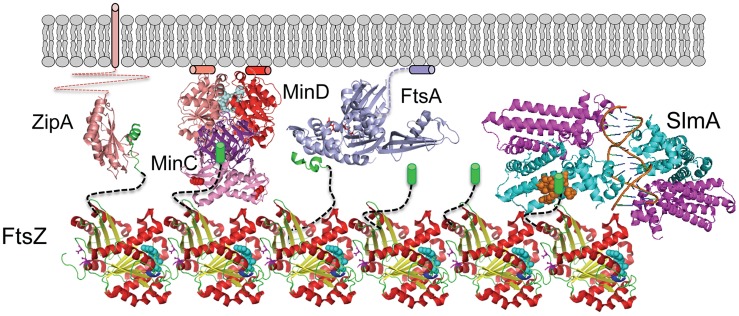
SlmA's mode of action shares features employed by MinCD. FtsZ (PDB: 1OFU) filaments are tethered to the membrane by interaction of the conserved tail of FtsZ with the membrane anchors ZipA (PDB:1F47) and FtsA (PDB:4A2A). The conserved tail of FtsZ (green helix or cylinder) is connected to the body of FtsZ by an unstructured linker (black dashed line). MinD (PDB:3Q9L) binds to the membrane and recruits MinC (PDB:1HF2), which interacts with the tail of FtsZ through the MinC^C^ domain and a second interaction mediated by the MinC^N^ domain. Binding to DNA activates SlmA (PDB: 4GCL) to bind the tail of FtsZ which we propose results in further interaction disrupting the FtsZ filament. The brown spheres indicate residues in SlmA that affect the binding of FtsZ (21) and may be where the tail binds.

This study confirms that SlmA, the effector of NO, antagonizes FtsZ assembly and demonstrates that the conserved tail of FtsZ is required for SlmA action. These results along with the SlmA resistance of the new FtsZ mutants suggests that the mechanism SlmA employs for disrupting FtsZ filaments is similar to the MinC/MinD mechanism (Fig. 8). In both cases, the inhibitors are activated by binding to a surface, SlmA binds to DNA and MinC/MinD to the membrane. Binding to a surface serves two purposes: 1) it positions the inhibitor within the cell, and 2) it leads to increased affinity for the tail of FtsZ, which puts the inhibitor in contact with the FtsZ filament resulting in disruption of the filament. Thus, two unrelated inhibitors, SlmA and MinC/MinD, use a similar mechanism to antagonize Z ring assembly.

### SlmA and the tail of FtsZ

Our finding that SlmA binds the tail of FtsZ was unexpected since it was reported that the tail of FtsZ was not involved in interaction with FtsZ [19]. However, after we failed to isolate FtsZ mutants defective in the binding of SlmA, a reexamination of the SlmA-FtsZ interaction revealed that the binding and activity of SlmA required the tail of FtsZ. We also demonstrated that ZipA competes with SlmA for the tail of FtsZ and that two of the more conserved residues in the tail, important for binding other partners of FtsZ, are required for the binding of SlmA. Thus, the conserved Z tail is required for interaction with at least six partners; FtsA, ZipA, MinC/MinD, ClpX, ZapD and SlmA.

Although we did not isolate FtsZ tail mutants resistant to SlmA in our screen, we previously isolated FtsZ tail mutants resistant to MinC/MinD. We observed that one out of four of these mutants (FtsZ-K380M) also conferred a moderate level of resistance to SlmA providing *in vivo* evidence for the importance of the interaction between the FtsZ tail and SlmA. Since only one of these mutants conferred resistance to SlmA it argues, that although both inhibitors bind the same Z tail peptide, they bind the tail differently. Perhaps the interaction of the tail with SlmA too closely mimics one of the essential interactions with ZipA or FtsA whereas the binding to MinC/MinD is different enough that resistant mutants can be isolated without compromising an essential activity.

The ability of SlmA to depolymerize FtsZ requires the tail of FtsZ which suggests that SlmA bound to DNA contacts FtsZ filaments anchored to the membrane. If so, SlmA bound to SBS sites must be at the periphery of the nucleoid so that it can interact with FtsZ filaments anchored to the membrane through interaction of the FtsZ tail with FtsA and ZipA. That some part of the nucleoid is in close proximity to the membrane is illustrated in *Vibrio cholerae* where the ToxR transcription regulator, an inner membrane protein, interacts directly with DNA to regulate the ToxR regulon [32].

### FtsZ mutants resistant to SlmA

The two FtsZ mutants we examined in detail were specifically resistant to SlmA as they displayed no resistance to SulA or MinC/MinD. Of the two, FtsZ-K190V was significantly more resistant than FtsZ-D86N. It not only displayed resistance to a higher level of SlmA but was synthetic sick with loss of Min. We do not know the basis of resistance for these mutants and suspect that the resistance of FtsZ-D86N may be indirect. Polymers of this mutant protein have an increased tendency to bundle *in vitro* as does FtsZ-D86K, which was reported earlier [33], [34]. We observed that this latter mutant also displays a level of resistance similar to FtsZ-D86N and it is possible that this ability to bundle confers resistance.

FtsZ-K190V confers significant resistance to SlmA. At the high levels of SlmA induction where the FtsZ-K190V mutant is killed, the killing is no longer dependent on the presence of the plasmid containing SBS sites and appears to be due to interference with DNA segregation as suggested earlier [17]. Interestingly, the K190 residue is located in the middle of the H7 helix, which is involved in conformational changes between the two sub-domains of the globular domain of FtsZ [35]. A mutation affecting the corresponding residue in *Staphylococcus aureus* FtsZ (R191P) confers resistance to the chemical inhibitor PC190723, although it is not involved directly in binding the inhibitor [35], [36]. One possibility is that SlmA stimulates a conformational change in FtsZ after binding to the tail of FtsZ by contacting a second region of FtsZ. If so, our mutant studies (an arginine substitution does not provide resistance) suggest that SlmA requires a positive charge at this residue of FtsZ to induce the change that destabilizes the filament. Evidence for a second interaction site between SlmA and FtsZ comes from SAXS studies where a weak interaction was detected between the globular domain of FtsZ and SlmA [19]. This interaction was independent of the tail of FtsZ, however, the resolution is too low to determine the residues in either protein that are involved.

### Model for SlmA action

The initial model for SlmA action proposed upon discovery of SlmA was that SlmA bound to the DNA could compete with the membrane anchors for FtsZ and strip FtsZ filaments off the membrane [7]. Our finding that SlmA binds the tail of FtsZ is consistent with such a model, however, several factors suggest such a model is not sufficient. The first is that the number of SlmA molecules (∼400) in the cell is quite limited compared to the amount of FtsZ (∼5000) and its anchors (FtsA ∼1000; ZipA ∼3500) and therefore unlikely to be sufficient to compete with the membrane anchors [7], [37]–[39]. The second is the *in vitro* data showing that SlmA causes disassembly of FtsZ filaments. The third is the existence of the FtsZ mutants that we isolated, particularly FtsZ-K190V, which do not affect the tail of FtsZ yet are resistant to SlmA. If SlmA only interacted with the tail of FtsZ one would not expect it to exert a negative effect on polymerization, since the tail is not involved in polymerization.

More recently, two models to account for SlmA action have been proposed. In one model SlmA bound to DNA activates SlmA to antagonize FtsZ assembly whereas in the second model FtsZ filaments bound to the SlmA/SBS complex are unable to assemble into a Z ring [17], [20]. This latter model was proposed because SlmA was not observed to antagonize FtsZ assembly. Our *in vitro* results confirmed the previous finding from the Bernhardt lab that SlmA antagonizes FtsZ assembly. Furthermore, our finding that the tail of FtsZ is essential for this activity, even though the tail has no direct role in FtsZ polymerization, along with the resistance of the FtsZ-K190V mutant, indicates a more active role for SlmA than simple competition for the tail or sequestration. In our model SlmA bound to DNA is at the periphery of the nucleoid and comes into contact with FtsZ filaments tethered to the membrane by FtsA and ZipA (Fig. 8). Upon binding to a tail within the FtsZ filament SlmA makes an additional contact with FtsZ inducing a conformational change leading to breakage of the filament. This catalytic behavior of SlmA results in destruction of the filament. It will be of interest to see if this mechanism is used by spatial regulators of the Z ring in other organisms.

## Materials and Methods

### Bacterial strains, plasmids, SBS molecules and growth conditions

Strains and plasmids used in this study are listed in Tables S5 and S6, respectively. Strains were grown in LB medium at 37°C unless otherwise indicated. When needed, antibiotics were used at the following concentrations: ampicillin = 100 µg/ml; spectinomycin = 25 µg/ml; kanamycin = 25 µg/ml; tetracycline = 25 µg/ml; and chloramphenicol = 20 µg/ml. The sequences of the various SBS DNA molecules are shown in Table S7.

The strain PS1603 (W3110 *slmA::cat*) was generated by *S. Pichoff* (unpublished) in which most of the *slmA* coding sequence was replaced with the *cat* gene expressing chloramphenicol resistance.

The Δ*min*Δ*slmA* double mutant strain DU5 (*W3110 min::kan*, *slmA::cat*) was generated by P1 transducing *slmA::cat* (from PS1603) into the strain *W3110 min::kan*. Chloramphenicol and kanamycin resistant transductants were selected at 42°C.

Strain DU11 (*W3110 ftsZ^0^ slmA<>frt recA::Tn10*)/pKD3C was constructed in several steps. First strain S3 (*W3110 leu::Tn10*) was transduced with P1 grown on TB85 (*MG1655*, *slmA::kan*) [7] and kanamycin resistant transductants were selected. The purified transductant was named DU8 (*W3110 leu::Tn10 slmA::kan*) and transformed with plasmid pCP20 by selecting ampicillin resistance at 30°C. The transformants were then streaked on LB plates with tetracycline and incubated at 42°C to get eliminate the *kan* gene and plasmid pCP20. The resultant strain was named DU9 (*W3110 leu::Tn10 slmA<>frt*) and confirmed by PCR using primers flanking the deleted region to confirm that the *kan* gene had been removed. Plasmid pKD3C was then transformed into DU9 and a purified transformant DU9/pKD3C was transduced with P1 grown on PB143 (*leu+ ftsZ^0^ recA::Tn10*) by selecting for Leu+ at 30° on M9 minimum medium. The resultant transductants were screened for temperature sensitivity and tetracycline resistance and the desired transductant (DU10/pKD3C) should have a genotype *leu+ ftsZ^0^ slmA<>frt* with plasmid pKD3C [40] providing FtsZ. Finally, the *recA::Tn10* allele from PB143 was transduced into DU10/pKD3C by selecting for tetracycline resistance on LB plates at 30°C. The resultant transductants were checked for UV sensitivity to confirm the inactivation of *recA* and the transductant was named DU11 (*W3110 leu+ ftsZ0 slmA<>frt recA::Tn10*)/pKD3C.

Strain SD139 and SD140 were constructed by transducing the *parC^TS^* allele from WM1033 (MG1655, *parC*281::Tn10) into strains W3110 and PS1603 respectively. The *parC^TS^* allele is linked to a Tn*10* and tetracycline resistant transductants obtained at 30°C were checked for the TS phenotype at 42°C.

Strains SD160, SD163 and SD164 were constructed similarly by replacing the *ftsZ84* allele on the chromosome of the parental strain PS106 with the *ftsZ-K190V*, *ftsZ-D86N* or *ftsZ-K190V&D86N* alleles using the lambda RED system [22]. PCR products of *ftsZ* fragments containing *ftsZ-K190V*, *ftsZ-D86N* or *ftsZ-K190V&D86N* mutations were electroporated into PS106/pKD46 induced with 0.04% arabinose for 3 hours at 30°C. The recombinants were selected on LB plates without salt at 42°C. 8 recombinants from the plates were randomly selected and then transformed with plasmid pSD133 and p2SBSK to check resistance to delocalized SBS bound SlmA. Recombinants resistant to SBS-SlmA were then checked for the presence of *ftsZ-K190V*, *ftsZ-D86N* or *ftsZ-K190V&D86N* by PCR and sequencing.

SD161 and SD165 were constructed simply by introduction of the *slmA::cat* allele from PS1603 into strain SD160 and SD163 by P1 transduction. Similarly, strains SD162 and SD167 were created by P1 transduction to introduce the *min::kan* allele from S4 into strain SD160 and SD163.

Strains SD170 and SD171 were created in two steps. The first step was to remove the *leu::Tn10* marker from SD160 and SD163 by transduction with P1 grown on PB143 (*leu+ ftsZ^0^ recA::Tn10*) and then selecting for Leu+ transductants at 37°C on M9 minimum medium. Transdcutants were tested for tetracycline sensitivity and resistance to SlmA in the presence of the multicopy plasmid p2SBSK (carries two SBS sites). The positive clones were named SD168 and SD169 and used as template for PCR to amplify the *ftsZ* gene and sequencing to make sure the colonies retained the *ftsZ-K190V* or *ftsZ-D86N* mutation. The second step was to introduce the *parC^TS^* allele into SD168 and SD169 obtained from the first step. SD168 and SD169 were transduced with P1 grown on WM1033 (*parC^TS^-Tn10*) and tetracycline resistance transductants were selected at 30°. The colonies obtained were purified and tested for temperature sensitivity at 42°C and were named SD170 and SD171.

pUC18K was constructed by replacing the *bla* gene in pUC18 with *kan*. To do this, an *Xho*I site was first introduced into pUC18 (pUC18blaX) at the end of *bla*, using primers XhoI-5′: 5′-GTAACTGTCAGACCAAGT*CTCGAG*ATATATACTTTAGATTG-3′ and XhoI-3′: 5′-CAATCTAAAGTATATAT*CTCGAG*ACTTGGTCTGACAGTTAC-3′. The *aph* coding sequence from pKNT25 was amplified by using primers Kan-5′-SspI: 5′-CAGT*AATATT*CTGATCAAGAGACAGGATGAG-3′ and Kan-3′-XhoI: 5′-CAGT*CTCGAG*CATTTCGAACCCCAGAG-3′. The PCR product was digested with SspI and XhoI and cloned into the same sites in puC18blaX (XhoI) to generate pUC18K. A derivative of pUC18K was made by introducing a fragment containing SBS12 and SBS17 to create p2SBSK. This fragment was obtained by PCR using primers SBS12-F-HindIII: 5′-GCAT*AAGCTT*GCGAAGTGAACGCTAACTCACATCTAACAATGCGCTCATCG-3′ and SBS17-R-EcoRI: 5′-GCAT*GAATTC*CGTTAGTGACCATTTACTTACTCAGGACGGGTGTGGTCGCCATG-3′. The resulting PCR fragment contains a segment of pBR322 sandwiched between the SBS12 and SBS17 sites, and was digested with EcoRI and HindIII and ligated into pUC18K cut with same enzymes.

Plasmid pSEB160 was created by S. Pichoff by inserting an SstI/HindIII digested fragment containing *ftsZ* into pBAD18 cut with the same enzymes (unpublished data). pSEB160 derivatives containing *ftsZ-360*, *ftsZ-K190V*, *ftsZ-D86N*, *ftsZ-I374K* and *ftsZ-L378E* were generated by site-directed mutagenesis.

Plasmid pSD119 was created by replacing the *ftsZ* coding sequence of pSEB160 with sequence encoding 6× His tagged *ftsZ* amplified from pSEB160 using primers His-FtsZ-SstI: 5′-TTC*GAGCTC*AGGCGACAGGCACAAATCGGAGAGAAATATG**CATCACCATCACCATCAC**TTTGAACCAATGGAACTTACC-3′ and His-FtsZ-HindIII: 5′-GCCAAAACAG*AAGCTT*CCTCGAAACCCAAATTCCAGTCAATTC-3′. The amplified fragment was digested with SstI and HindIII and ligated to pSEB160 digested with the same enzymes. pSD119 derivatives for 6× His-*ftsZ*-320 and 6× His-*ftsZ*-360 expression were created by site directed mutagenesis by addition of two stop codons after codons FtsZ320 and FtsZ360. Primers used are FtsZ320: 5′-GGCATGGACAAACGT**TGATAA**ATCACTCTGGTGACC-3′ and FtsZ360: 5′-GCTAAAGTCGTGAATGAC**TGATAA**CCGCAAACTGCGAAAG-3′.

The plasmid pSD128 was constructed by inserting an *Eco*RI and *Hin*dIII fragment containing the *slmA* coding sequence into the *Eco*RI and *Hin*dIII double digested pBANG59. The *slmA* containing fragment was amplified from chromosomal DNA using primers slmA-5′-EcoRI: 5′-AGT*GAATTC*TTTC**AGGAGG**ATAATGTAACATGGCAGAAAAACAAACTG-3′ and slmA-3′-HindIII: 5′-GCG*AAGCTT*TTGGCGTTTAAAGAAACTC-3′. The ribosome binding site for *slmA* translation was changed to the consensus sequence –**AGGAGG-** through this approach. The pSD128 derivatives containing different mutations were constructed by site-directed mutagenesis.

Plasmid pSD133 containing *slmA* with its own ribosome binding site is otherwise similar to pSD128 and was constructed in a similar manner, but the *slmA* containing fragment was amplified from chromosomal DNA using different primer pairs: slmA-For: 5′-CGT*GAATTC*CGCCTGGCAAGTGCTTA-3′ and slmA-3′-HindIII.

The plasmid pQE80-*slmA* was created by ligation of a *Bam*HI-*Pst*I fragment containing the *slmA* coding sequence and pQE80 (Qiagen) cut with the same enzymes. The fragment was amplified from chromosomal DNA using primers 5′-6×his-slmA: 5′-GT*GGATCC*GCAGAAAAACAAACTGCG-3′ and ttk-PstI-3′: 5′-GAAA*CTGCAG*CGGCGTCATATTACTGC-3′. Site-directed mutagenesis was used to introduce different *slmA* mutations into pQE80-*slmA* to obtain various derivatives.

The plasmid pSD198 was created by ligation of a *Bam*HI-*HindIII* fragment containing the *zipA_185–328_* coding sequence and pQE80 (Qiagen) cut with the same enzymes. The fragment was amplified from chromosomal DNA using primers zipA185-5′-BamHI: 5′- GACT*GGATCC*GATAAACCGAAGCGCAAAG -3′ and zipA-3′-HindIII: 5′- GACT*AAGCTT*GGTTCGAAGAGGAGTTAAT-3′.

Plasmids pSlmA-T25 and pSlmA-T18 were constructed by inserting a *Bam*HI/*Hin*dIII cut fragment containing the *slmA* coding sequence into the vectors pKNT25 and pUT18, respectively, cut with the same enzymes. The fragment was amplified from chromosomal DNA using the primer pair slmA-BTHN-BamHI: 5′- GTC*GGATCC*TGCAACTGTGCCGCAAT-3′ and slmA-BTHN-HindIII: 5′- TGT*AAGCTT*GGCAGAAAAACAAACTG-3′. Derivatives of pSlmA-T25 and pSlmA-T18 containing *slmA* mutations were created by site-directed mutagenesis. Plasmids pZT25 and pZT18 were made by inserting a *Bam*H1/*Hin*dIII fragment containing the *ftsZ* coding sequence into pKNT25 and pUT18, respectively. Derivatives of these plasmids containing various *slmA* mutations or *ftsZ* mutations were created by site-directed mutagenesis.

Plasmid pSUMO-SlmA was constructed by ligation of a BsaI-XbaI fragment containing the *slmA* coding sequence and pE-SUMO-amp (LiferSensors) cut with the same enzyme. The fragment was amplified from plasmid pSD133 using primers slmA-SUMO-F: 5′-ACGT*GGTCTC*GAGGTGCAGAAAAACAAACTGCGAAAAG-3′ and SlmA-SUMO-R: 5′-CAGT*TCTAGA*GTCATCCGGCGTCATATTAC-3′.

### Creation of the functional FtsZ mutant libraries and selection for SBS-SlmA resistant *ftsZ* mutations

The procedure was carried out as previously described for selection of MinCD resistant FtsZ mutants [15]. PCR random mutagenesis was used to introduce random mutations into the coding region of *ftsZ* gene using pBANG112 as the template and primers: 5′- GCCTCAGGCGACAGGCACAAATCGGAGAG and 5′-GCTGCAGATATTCGATATCACGCATGAAAC. The purified PCR fragments were then digested with EcoRI and EagI and ligated into pBANG112 digested with the same enzymes. The ligation product was then electroporated into DU11/pKD3C &pSD133 and transformants selected at 42°C on LB plates with ampicillin. All colonies that grew were pooled together and part of the pooled culture was subjected to plasmid extraction to make a stock of the FtsZ mutant library. To select for the SBS-SlmA resistant FtsZ mutants, the rest of the pooled cells was transformed with plasmid p2SBSK and colonies resistant to delocalized SBS-SlmA were selected with 20 µM IPTG at 30°C on plates containing ampicillin, spectinomycin and kanamycin. Plasmids were isolated from the colonies that grew up and the *ftsZ* gene in the plasmids was sequenced to identify the mutations.

### Bacterial two-hybrid assay

To detect SlmA-FtsZ and SlmA-SlmA interactions, appropriate plasmid pairs encoding FtsZ-T18 and SlmA-T25 or FtsZ-T18 and SlmA-T25 or their variants were co-transformed into BTH101 [41]. Single colonies were resuspended in 1 ml LB medium and 3 µl of each aliquot was spotted on LB plates containing 100 µg/ml ampicillin, 25 µg/ml kanamycin, 40 µg/ml X-gal and 250 µM IPTG. Plates were incubated at 30°C for 36 hours before analysis.

### Protein purification

His-SlmA and variants containing different mutations were expressed and purified from JS238/pQE80-*slmA* and its derivatives following the protocol used to purify 6×his-ZapA [23]. An overnight culture of each strain grown in LB with ampicillin (100 µg/ml) and glucose (0.2%) was diluted 1∶100 into 1 L fresh LB medium supplemented with ampicillin (100 µg/ml) and incubated at 37°C until OD_540_ reached about 0.4. IPTG was then added to the culture to a final concentration of 1 mM and incubated at 37°C for another 3 hours. Cells were collected by centrifugation, washed with 10 mM Tris-HCl (pH 7.9), and frozen at −80°C until used. On the day of purification, the cells were thawed and resuspended in 20 ml lysis buffer (20 mM Tris-HCl [pH 7.9], 70 mM NaCl and 20 mM imidazole) and passed through the French press twice (10,000 psi). The lysates were centrifuged at 12,000 rpm for 15 min at 4°C to remove cell debris. The supernatants were removed and loaded onto pre-equilibrated Ni-NTA resin (Qiagen). The column was washed once with high salt wash buffer (20 mM Tris-HCl [pH 7.9], 500 mM NaCl and 20 mM imidazole) and once with the same buffer except with the imidazole concentration increased to 50 mM. The bound protein was eluted with elution buffer (20 mM Tris-HCl [pH 7.9], 500 mM NaCl and 250 mM imidazole). The peak fractions were dialyzed against the storage buffer (25 mM Tris-HCl [pH 7.9], 200 mM KCl, 1 mM EDTA and 10% glycerol) overnight and stored at −80°C until use.

The untagged version of SlmA was expressed and purified from BL21 (λDE3)/pLysS cells containing pSUMO-SlmA. Purification of the H-SUMO-SlmA fusion protein was similar to purification of the 6×his-SlmA. After dialysis, the H-SUMO tag was cleaved with purified 6×His-tagged SUMO protease (Ulp1) for 1 hour at 30°C in the protein storage buffer (25 mM Tris-HCl [pH 7.9], 200 mM KCl, 1 mM EDTA and 10% glycerol) with 1 mM DTT. The released tag and protease were removed by passing it through the pre-equilibrated Ni-NTA resin. Untagged SlmA was collected in the flow through, concentrated and stored at −80°C.

N-terminal 6×His-tagged FtsZ, FtsZ320 and FtsZ360 were purified from JS238 cells containing plasmids pSD119, pSD119-Z320 or pSD119-Z360 respectively. An overnight culture of each strain grown in LB with ampicillin (100 µg/ml) and glucose (0.2%) was diluted 1∶100 into 1 L fresh LB medium supplemented with ampicillin (100 µg/ml) and incubated at 37°C until OD_540_ reached about 0.4. Arabinose was then added to the culture to a final concentration of 0.2% and incubated at 37°C for another 3 hours. Cells were collected by centrifugation, washed with 10 mM Tris-HCl (pH 7.9), and frozen at −80°C until used. The subsequent procedures were similar to purification of 6×his-SlmA.

Induction of wild type FtsZ, FtsZ-K190V and FtsZ-D86N was similar to induction of 6×His-tagged FtsZ in JS328 cells containing pSEB160, pSEB160-360, pSEB160-K190V, pSEB160-D86N, pSEB160-I374K and pSEB160-L378E respectively. After collecting the cells, FtsZ-WT, FtsZ-K190V and FtsZ-D86N as well as the other mutant proteins were purified according to the procedure described previously [15], [42].

### FtsZ polymerization and electron microscopy

FtsZ polymerization reactions were in Pol buffer (50 mM HEPES-NaOH [pH 8.0], 200 mM KCl and 10 mM MgCl_2_). The SBS17 fragment (30 bp) and SlmA or SlmA mutants were mixed together in a separate tube and incubated at room temperature for 10 minutes before addition to the polymerization reactions. Unless specified, the SlmA used was His tagged SlmA. The SBS17 probe used here was prepared by annealing two un-labeled complementary 30 base oligonucleotides SBS17-F and SBS17-R. FtsZ was added to a final concentration of 2 µM in a 50 µl reaction containing pre-formed SBS17-SlmA, or SBS17 alone, or SlmA or just DNA binding buffer. After 5 min incubation, GTP or GMPCPP was added to a final concentration of 1 mM and incubation at room temperature continued for another 5 min before the samples were loaded onto grids. 15 µl of 1% uranyl acetate was spotted on the grid for 1 min and blotted away. The grids were air-dried and imaged with a JEOL-JEM-1400 transmission electron microscope.

The co-sedimentation assay was performed similarly as above except that the protein concentrations were 5 µM. After the addition of GMPCPP, the reactions were subjected to ultracentrifugation at 80,000 rpm for 15 min at 25°C in TLA100.2 rotor and a Beckman TL-100 centrifuge. Supernatants and pellets were then analyzed by SDS-PAGE.

### Biolayer interferometry assays

The assays were performed in 250 µl of 1× Pol buffer (50 mM HEPES-NaOH [pH 8.0], 200 mM KCl, 10 mM MgCl_2_) using a biosensor system (FortéBio) at room temperature. The biotinylated SBS17 probe was prepared by annealing two complementary 30 base oligonucleotides, the biotinylated-SBS17-F and SBS17-R. FtsZ and SlmA proteins were diluted in 1× FtsZ polymerization buffer before the test. To measure the binding affinity of SlmA variants for the biotinylated SBS17 probe, streptavidin-coated biosensors tips were equilibrated with 1× Pol buffer to establish a baseline prior to biotinylated SBS17 immobilization. 250 µL of 1× Pol buffer containing 50 nM biotinylated SBS17 was incubated with the biosensor tips with shaking at 2,200 rpm for 5 minutes. After the immobilization, the biosensor tips were washed with 1× Pol buffer for 10 seconds. Association of SlmA-WT or SlmA mutants to the biosensors was monitored for 2 minutes in 250 µl 1× Pol buffer containing 4 µM SlmA with agitation at 2,200 r.p.m. Dissociation was initiated by dipping the biotinylated SBS17-SlmA coated biosensor tips into 250 µl of 1× Pol buffer, and the process was monitored continuously for 2 minutes while agitating at 2,200 rpm. Data were obtained automatically by the biosensor User Software (FortéBio) and were subsequently analyzed by global fitting using the GraphPad Prism 5 software.

Binding of FtsZ to the biotinylated-SBS17-SlmA complex was performed similarly as above. After association of SlmA-WT or SlmA mutant to the biotinylated SBS17 coated biosensor tips, the tips were washed with 250 µl of 1× Pol buffer for 10 seconds. FtsZ was preincubated with 1 mM GDP for 5 minutes before the SBS17-SlmA complex coated biosensor tips were dipped into the solution. ZipA*_185–328_* was added at different concentrations with GDP to see whether it blocked FtsZ binding to SBS-bound SlmA. Association was monitored for 1 minute in 250 µl of 1× FtsZ Pol buffer containing 4 µM FtsZ with agitation at 2,200 rpm followed by dissociation in the same buffer without FtsZ for 2 minutes. Data were collected and analyzed with Graphpad Prism 5.

In a reciprocal approach, 6×His-tagged FtsZ-FL, FtsZ320 or FtsZ360 was immobilized at the surface of Ni-NTA biosensors and untagged SlmA preincubated with or without SBS DNA was tested for binding to 6×His-tagged FtsZ variants. In these assays, 250 µL of 1× Pol buffer containing 1 µM 6×His-tagged FtsZ mutant was incubated with the biosensor tips with shaking at 2,200 r.p.m for 5 minutes. After the immobilization, the biosensor tips were washed with 1× Pol buffer for 10 seconds. Association of untagged SlmA preincubated with or without SBS DNA to the biosensors was monitored for 2 minutes in 250 µl 1× Pol buffer with agitation at 2,200 r.p.m. Dissociation was initiated by dipping the FtsZ-SBS-SlmA coated biosensor tips into 250 µl of 1× Pol buffer, and the process was monitored continuously for 2 minutes while agitating at 2,200 r.p.m. Data were generated automatically by the BLItz User Software version and were subsequently analyzed by global fitting using the GraphPad Prism 5 software.

## Supporting Information

Figure S1 Mutations in two different regions of FtsZ confer resistance to de-localized SlmA. A spot test for resistance of FtsZ mutants to the de-localized SBS-SlmA. Plasmids pSD133 (P*tac::slmA*) and p2SBSK (pUC18 with 2SBS sites) were introduced into the *ftsZ^−^* strain DU11 (*ftsZ^0^ slmA<frt>recA::Tn10*) complemented with pBANG112 or its derivatives containing different *ftsZ* alleles. One colony of each resultant strain was resuspended in 1 ml LB medium, serially diluted by 10 and 3 µl from each dilution was spotted on LB plates containing various concentrations of IPTG and supplemented with antibiotics. The plates were incubated at 30°C for 30 hours before being photographed. The vector pUC18K was used as control for resistance to the overproduction of SlmA.
(EPS)

Figure S2 Characterization of FtsZ-K190V, FtsZ-D86N and FtsZ-K190V&D86N mutant strains. A) Morphology of *S3(W3110 ftsZ-WT)*, *SD 160 (W3110, ftsZ-K190V)*, *SD163 (W3110 ftsZ-D86N)*, *and SD164 (W3110, ftsZ-D86N&K190V)* growing at different temperatures. Overnight cultures of the indicated strains grown at 42°C were diluted 100 times in LB supplemented with antibiotics and incubated at 42°C. At OD_540_ = 0.3, the cultures were diluted 10 fold in fresh LB medium, split into three parts, and grown at 42°C, 37°C and 30°C for an additional 2 hours before photographing. B) Growth of the strains in (A).
(EPS)

Figure S3 The FtsZ-K190V and FtsZ-D86N mutants display resistance to overproduction of SlmA-T33A. A) A spot test to determine the resistance of FtsZ-K190V and FtsZ-D86N to overproduction of SlmA-T33A (DNA binding mutant). Strains SD110 (*slmA::cat*), SD161 (*ftsZ-K190V slmA::cat*) and SD165 (*ftsZ-D86N slmA::cat*) were transformed with plasmid pSD128-T33A. Single colonies were picked into 1 ml LB, serially diluted 10 fold and spotted on plates with increasing IPTG concentrations. B) Morphology of cells in (A) growing in liquid LB medium with 100 µM IPTG.
(EPS)

Figure S4 FtsZ-K190V and FtsZ-D86N mutants do not confer resistance to other division inhibitors. A) Spot test to determine the resistance of FtsZ-K190V and FtsZ-D86N to SulA. Strains containing the indicated *ftsZ* mutations (SD160 [*ftsZ-K190V*] and SD163 [*ftsZ-D86N*]) along with the wild type control (S3) were transformed with the SulA expression plasmid pBS31. A single colony of each resultant strain was then resuspended in 1 ml LB medium, diluted serially by 10 and 3 µl of each dilution was spotted on LB plates supplemented with ampicillin (pBS31) and various IPTG concentrations. The plates were then incubated at 37°C overnight before photographing. B) Effect of MinCD expression on the morphology of strains carrying the indicated *ftsZ* mutations. Strains carrying the *ftsZ* mutations (SD160 [*ftsZ-K190V*] and SD163 [*ftsZ-D86N*]) along with the wild type control (S3) were transformed with the MinCD expression plasmid pBANG59. Overnight cultures of each of the indicated strains were diluted 100 fold in LB medium supplemented with spectinomycin with or without IPTG and then grown at 37°C for 2 hours before photography.
(EPS)

Figure S5 FtsZ-K190V but not FtsZ-D86N is synthetic sick with a *min* deletion. A) Spot test to examine the synthetic lethality of strains S4 (*W3110 min::kan*), DU5 (*W3110 min::kan slmA::cat*), SD162 (*W3110 ftsZ-K190V min::kan*) and DU167 (*W3110 ftsZ-D8N min::kan*). A single colony of each strain grown on LB plates at 42°C was resuspended in 1 ml LB medium and serially diluted by 10. 3 µl of each dilution was then spotted on two LB plates supplemented with kanamycin, one of which was incubated at 42°C overnight and the other was incubated at 30°C for 20 hours. B) Morphology of the above strains after being shifted to 30°C for 2 and half hours. Exponentially growing cultures of the strains in at 42°C were diluted 10 fold and grown at 30°C for 2 and half hours before photography.
(EPS)

Figure S6 SlmA is activated by binding to SBS DNA to antagonize FtsZ polymerization. A) FtsZ polymerization with or without the addition of SlmA and SBS17-30mer in Cho's buffer (50 mM PIPES [pH 6.7], 10 mM MgCl_2_ and 200 mM KCl) and Tonthat's buffer (50 mM HEPES [pH 7.7], 5 mM MgCl_2_ and 100 mM Kacetate). Reactions were performed in 50 µl volume containing FtsZ (2 µM) and GTP (1 mM) with or without the addition of SlmA (2 µM) and SBS17-30mer DNA (2 µM). Reactions were incubated at room temperature for 5 minutes and then samples were spotted onto carbon-coated grids for 1 minute and negatively stained with 1% uranyl acetate and visualized by electron microscopy. Left and middle panels were performed in Cho's buffer while right panel was performed in Tonthat's panel. B) FtsZ polymerization with or without the addition of SlmA and SBS DNA of different lengths in Du's buffer (50 mM HEPES, pH 8.0, 10 mM MgCl_2_ and 200 mM KCl). Reactions were performed as in (A). Scale bar is 100 nm. C) Assessment of SlmA bound to SBS of varying lengths to bundle stable FtsZ filaments. Reactions were performed in 50 µl volume containing FtsZ (2 µM) and GMPCPP (1 mM) with or without the addition of SlmA (2 µM) and SBS17DNA (2 µM) of varying lengths. All DNA fragments contained the SBS17 site in the center of the fragment. Samples were analyzed by negative stain electron microscopy after 5 minute incubation at room temperature.
(EPS)

Figure S7 SlmA and SBS17 do not affect the assembly of FtsZ or FtsZ mutants when added alone. Reactions were performed in 50 µl volume containing FtsZ or FtsZ mutants (2 µM) and GTP (1 mM) in the absence of any additions or in the presence of SlmA (2 µM) or SBS17-30mer DNA (2 µM). After incubation for 5 minutes at room temperature the samples were analyzed by negative stain electron microscopy.
(EPS)

Figure S8 FtsZ-K190V and FtsZ-D86N still interact with SlmA. A) Bacterial two-hybrid assay to analyze the interaction between SlmA and FtsZ or FtsZ mutants. Plasmid pairs, as indicated, were co-transformed into BTH101. Individual colonies were resuspended in 1 ml LB medium and spotted on LB plates supplemented with antibiotics and 40 µg/ml X-gal and 250 µM IPTG. Plates were incubated at 30°C for 36 hours before taking pictures. B) SBS17-30mer bound SlmA co-sediments with stable mutant FtsZ polymers formed with GMPCPP. Polymerization assays were done with a protein concentration of 5 µM and GTP replaced by GMPCPP (1 mM). SBS17-30mer was kept at 2 µM. After the reactions were incubated at room temperature for 5 minutes, FtsZ polymers were sedimented by ultracentrifugation. Proteins in the supernatant and pellet fractions were separated by SDS-PAGE gel. C) SBS17-30mer bound SlmA did not sediment in the absence of FtsZ-GMPCPP polymers. Reactions were performed as B). D) SBS17-30mer bound SlmA causes bundling of the FtsZ mutants assembled with GMPCPP. Samples from reactions analogous to those in (B) were examined by negative stain electron microscopy. Scale bar is 100 nm.
(EPS)

Figure S9 Biolayer interferometry assay to assess SlmA binding to DNA and FtsZ. A) Streptavidin biosensor tips were loaded with 50 nM biotinylated SBS17-30mer for 5 minutes followed by a 10 second wash. Association was initiated by moving the SBS17-30mer coated tips to tubes containing 4 µM SlmA or SlmA mutants. Two minutes after incubation, the tips were moved to tubes containing FtsZ polymerization buffer and dissociation monitored for 2 minutes. The DNA binding mutant SlmA-T33A was used as a control. B) SlmA-WT interacts with FtsZ. SBS17-30mer-SlmA complexes were generated as in (A). The tips were then moved to tubes containing 4 µM FtsZ to measure the association for 1 minute and then into buffer lacking FtsZ to follow dissociation for 2 minutes (dissociation step is not shown). SlmA-R73D and SlmA-F65A, which do not interact with FtsZ, were used as controls. C) SlmA bound to SBS DNA of different lengths binds to 6×His-FtsZ. Ni-NTA biosensor tips were loaded with 1 µM 6×His-FtsZ for 5 minutes followed by a 10 second wash. Association was initiated by moving the 6×His-FtsZ coated tips to tubes containing SlmA (untagged version, 1 µM) with or without SBS DNA of various lengths (1 µM) preincubated for 10 minutes. Two minutes after the incubation, the tips were moved to tubes containing FtsZ polymerization buffer to monitor the dissociation for 2 minutes. D) SlmA-SBS binds with similar kinetics to His-FtsZ and His-FtsZ-K190V. Ni-NTA biosensor tips were loaded with 1 µM 6×His-FtsZ or 6×His-FtsZ-K190V for 5 minutes followed by a 10 second wash. Association was initiated by moving the tips to tubes containing SlmA (untagged version, 1 µM) and SBS17-30mer DNA (1 µM).
(EPS)

Figure S10 FtsZ mutants resistant to MinC^N^MinD are not resistant to SlmA. A) SAXS model of SlmA-FtsZ_316_ complex [19]. Based on this model, the H10 helix (colored blue) is at the interaction interface. Residues in the H10 helix whose substitutions confer resistance to MinC^N^/MinD are colored green. The two residues K190 and D86, mutations at which provide resistance to SlmA, are colored yellow and red, respectively. B) Examination of FtsZ mutants resistant to the N-terminal domain of MinC for resistance to de-localized SlmA. Plasmids pSD133 (P*tac::slmA*) and p2SBSK (pUC18 with 2SBS sites) were introduced into the *ftsZ^−^* strain DU11 (*ftsZ^0^ slmA<frt>recA::Tn10*) complemented with pBANG112 or its derivatives containing different *ftsZ* alleles. One colony of each resultant strain was resuspended in 1 ml LB medium, serially diluted by 10 and 3 µl from each dilution was spotted on LB plates containing various concentrations of IPTG and supplemented with ampicillin, spectinomycin and kanamycin. The plates were incubated at 30°C for 30 hours before photography. Helix 10 is colored blue and substitutions of residues resistant to the N-terminal domain of MinC are colored green. K190 and D86 are colored as yellow and red respectively.
(EPS)

Figure S11 The conserved tail of FtsZ is required for SlmA/SBS induced bundling of FtsZ filaments. His-FtsZ or his-FtsZ_360_ (2 µM) were incubated with 2 mM GMPCPP in the presence of absence of SlmA (2 µM) bound to SBS17-30mer (2 µM). After two minute incubation at room temperature the samples were spotted on grids, stained with uranyl acetated and examined by electron microscopy.
(EPS)

Table S1 Properties of FtsZ mutants examined in this study.
(DOCX)

Table S2 Average cell length of indicated strains grown at 42°C and 30°C.
(DOCX)

Table S3 GTPase activity of FtsZ mutants at two different salt concentrations.
(DOCX)

Table S4 Binding affinities of FtsZ to SlmA bound to DNA.
(DOCX)

Table S5 List of strains used in this study.
(DOCX)

Table S6 List of plasmids used in this study.
(DOCX)

Table S7 List of SBS DNA molecules used in this study.
(DOCX)
