# Supplementary material for: SlmA Antagonism of FtsZ Assembly Employs a Two-pronged Mechanism like MinCD
Source: PLoS Genet. 2014 Jul 31;10(7):e1004460. doi: 10.1371/journal.pgen.1004460 (PMC4117426; doi:10.1371/journal.pgen.1004460)
Supplement: Table S1 — Properties of FtsZ mutants examined in this study. (DOCX) [file pgen.1004460.s012.docx]

Table S1. Properties of FtsZ mutants examined in this study.

| FtsZ alleles | Complementation | Resistance to SBS-SlmA | Resistance to MinCD | Interaction with SBS-SlmA *in vitro* | Source |
| --- | --- | --- | --- | --- | --- |
| WT | YES | NO | NO | YES | Collection |
| K190I | YES | YES | NO | ND | This study |
| K190V | YES | YES | NO | YES | This study |
| K190L | YES | YES | ND | ND | This study |
| K190A | YES | YES | ND | ND | This study |
| K190N | YES | YES | ND | ND | This study |
| K190E | YES | YES | ND | ND | This study |
| K190W | YES | YES | ND | ND | This study |
| K190R | YES | NO | ND | ND | This study |
| D86N | YES | YES | NO | YES | This study |
| D86V | YES | NO | ND | ND | This study |
| D86K | YES | YES | ND | ND | This study |
| D86N& K190V | YES (≥37 °C) | YES | ND | ND | This study |
| L270V | YES | NO | YES (MinC^N^D) | ND | [[13](#_ENREF_2),1[4](#_ENREF_4)] |
| R271G | YES | NO | YES (MinC^N^D) | ND |  |
| E276D | YES | NO | YES (MinC^N^D) | ND |  |
| N280D | YES | NO | YES (MinC^N^D) | ND |  |
| D373E | YES | NO | YES (MinC^c^D) | ND |  |
| I374V | YES | NO | YES (MinC^c^D) | ND |  |
| A376P | YES | NO | YES (MinC^c^D) | ND |  |
| L378V | YES *(∆min or ∆slmA*) | ND | YES (MinC^c^D) | ND |  |
| K380M | YES | YES | YES (MinC^c^D) | ND |  |
| I374K | NO | ND | ND | NO | This study |
| L378E | NO | ND | ND | NO | This study |
| 320 | NO | ND | ND | NO | This study |
| 360 | NO | ND | ND | NO | This study |
| K190I&T57M | YES | YES | ND | ND | This study |
| K190I&A101D | YES | YES | ND | ND | This study |
| K190I&K380T | YES | YES | ND | ND | This study |
| D86N&G95D | YES | YES | ND | ND | This study |
| D86N&S246Y&M344I | YES | YES | ND | ND | This study |
| G95D | YES | NO | ND | ND | This study |
| S246Y | YES | NO | ND | ND | This study |
| M344I | YES | NO | ND | ND | This study |
