# Supplementary material for: SlmA Antagonism of FtsZ Assembly Employs a Two-pronged Mechanism like MinCD
Source: PLoS Genet. 2014 Jul 31;10(7):e1004460. doi: 10.1371/journal.pgen.1004460 (PMC4117426; doi:10.1371/journal.pgen.1004460)
Supplement: Table S2 — Average cell length of indicated strains grown at 42°C and 30°C. (DOCX) [file pgen.1004460.s013.docx]

Table S2. Average cell length of indicated strains grown at 42°C and 30 °C.

|  | Strain | Total cells | Average cell length (µm) |
| --- | --- | --- | --- |
| 42°C | Z-WT *∆min* | 216 | 7.2±5.6 |
|  | Z-WT *∆min ∆slmA* | 301 | 6.0±3.6 |
|  | Z-K190V ∆min | 229 | 6.8±4.1 |
|  | Z-D86N ∆min | 235 | 7.8±4.3 |
| 30°C | Z-WT *∆min* | 292 | 10.9±7.2 |
|  | Z-WT *∆min ∆slmA* | 208 | 26.8±12.5 |
|  | Z-K190V ∆min | 209 | 16.7±10 |
|  | Z-D86N ∆min | 227 | 10.0±5.3 |
