# Supplementary material for: SlmA Antagonism of FtsZ Assembly Employs a Two-pronged Mechanism like MinCD
Source: PLoS Genet. 2014 Jul 31;10(7):e1004460. doi: 10.1371/journal.pgen.1004460 (PMC4117426; doi:10.1371/journal.pgen.1004460)
Supplement: Table S3 — GTPase activity of FtsZ mutants at two different salt concentrations. (DOCX) [file pgen.1004460.s014.docx]

Table S3. GTPase activity of FtsZ mutants at two different salt concentrations.

| FtsZ | GTPase activity at 50 mM KCl (GTP/min/FtsZ) | GTPase activity at 200 mM KCl (GTP/min/FtsZ) |
| --- | --- | --- |
| FtsZ-WT | 4.6 | 9.8 |
| FtsZ-K190V | 4.0 | 4.4 |
| FtsZ-D86N | 3.8 | 5.3 |
