# Supplementary material for: SlmA Antagonism of FtsZ Assembly Employs a Two-pronged Mechanism like MinCD
Source: PLoS Genet. 2014 Jul 31;10(7):e1004460. doi: 10.1371/journal.pgen.1004460 (PMC4117426; doi:10.1371/journal.pgen.1004460)
Supplement: Table S4 — Binding affinities of FtsZ to SlmA bound to DNA. (DOCX) [file pgen.1004460.s015.docx]

Table S4.Binding affinities of FtsZ to SlmA bound to DNA.

| Binding affinities of SlmA mutants for the biotinylated SBS17-30mer | | | | |
| --- | --- | --- | --- | --- |
|  | *K*_D_ (M) | *k*_on_ [1/(M*s)] | *k*_off_ (1/s) | *R*^2^ |
| SlmA-WT | 6.29×10^-8^ | 3.24×10^+4^ | 2.04×10^-3^ | 0.98 |
| SlmA-F65A | 11.44×10^-8^ | 2.48×10^+4^ | 2.83×10^-3^ | 0.98 |
| SlmA-R73D | 10.63×10^-8^ | 2.31×10^+4^ | 2.46×10^-3^ | 0.98 |
| SlmA-T33A | No binding | | | |
| Binding affinities of SlmA and SBS-SlmA for 6×His-FtsZ | | | | |
| SlmA | 210×10^-7^ | 0.71×10^+4^ | 14.9×10^-2^ | 0.97 |
| SlmA + SBS17-30mer | 2.10×10^-7^ | 7.40×10^+4^ | 1.55×10^-2^ | 0.98 |
| SlmA + SBS17-20mer | 7.89×10^-7^ | 1.81×10^+4^ | 1.43×10^-2^ | 1.0 |
| SlmA + SBS-18mer | 3.09×10^-7^ | 2.16×10^+4^ | 0.67×10^-2^ | 1.0 |
| SlmA + SBS-14mer | 2.06×10^-7^ | 2.75×10^+4^ | 0.57×10^-2^ | 0.98 |

R^2^ is the coefficient of determination estimating the goodness of a curve fit reported by the GraphPad Prism 5.0 s software.
