# Supplementary material for: SlmA Antagonism of FtsZ Assembly Employs a Two-pronged Mechanism like MinCD
Source: PLoS Genet. 2014 Jul 31;10(7):e1004460. doi: 10.1371/journal.pgen.1004460 (PMC4117426; doi:10.1371/journal.pgen.1004460)
Supplement: Table S5 — List of strains used in this study. (DOCX) [file pgen.1004460.s016.docx]

Table S5. List of strains used in this study.

| Strain | Description | Source/reference |
| --- | --- | --- |
| BTH101  DU5  DU11/pKD3C  JS238  PS106  PS1603  S3  S4  S7/pKD3C  SD110  SD139  SD140  SD160  SD161  SD162  SD163  SD164  SD165  SD167  SD170  SD171  W3110  WM1033 | F- *cya-99, araD139 galE15 galK16 rpsL1 (Str^R^) hsdR2 mcrA1 mcrB1*    *W3110 leu::Tn10 min::kan slmA::cat*  *W3110 ftsZ^0^ slmA<> frt recA::Tn10*  MC1061 *malPp::lacI^q^ srlC::Tn10 recA1*  *W3110 leu::Tn10 ftsZ84*  *W3110 slmA::cat*  *W3110 leu::Tn10*  *W3110 leu::Tn10 min::kan*  *W3110 ftsZ^0^ slmA<frt> recA::Tn10*  *W3110 leu::Tn10 slmA::cat*  *W3110 parC281::Tn10*  *W3110 parC281::Tn10 slmA::cat*  *W3110 leu::Tn10 ftsZ-K190V*  *W3110 leu::Tn10 ftsZ-K190V slmA::cat*  *W3110 leu::Tn10 ftsZ-K190V min::kan*  *W3110 leu::Tn10 ftsZ-D86N*  *W3110 leu::Tn10 ftsZ-K190V&D86N*  *W3110 leu::Tn10 ftsZ-D86N slmA::cat*  *W3110 leu::Tn10 ftsZ-D86N min::kan*  *W3110 parC281::Tn10 ftsZ-K190V*  *W3110 parC281::Tn10 ftsZ-D86N*  *F^-^ λ^-^ rph-1 INV(rrnD, rrnE)*  *MG1655, parC281::Tn10* | [[41](#_ENREF_1)]  This study  This study  Lab collection  Lab collection  Lab collection  [[15](#_ENREF_2)]  [[15](#_ENREF_2)]  [[15](#_ENREF_2)]  This study  This study  This study  This study  This study  This study  This study  This study  This study  This study  This study  This study  Lab collection  W. Margolin (unpublished) |
