# Supplementary material for: SlmA Antagonism of FtsZ Assembly Employs a Two-pronged Mechanism like MinCD
Source: PLoS Genet. 2014 Jul 31;10(7):e1004460. doi: 10.1371/journal.pgen.1004460 (PMC4117426; doi:10.1371/journal.pgen.1004460)
Supplement: Table S6 — List of plasmids used in this study. (DOCX) [file pgen.1004460.s017.docx]

Table S6. List of plasmids used in this study.

| Plasmid | Description | ori | Source/reference |
| --- | --- | --- | --- |
| pBANG59  pBANG112  pBS31  pKD3C  pKNT25  pQE80-slmA  p2SBSK  pSEB160  pSD119  pSD128  pSD133  pSD198  pSlmA-T25  pSlmA-T18  pSUMO-SlmA  pUC18K  pUT18  pZT25  pZT18 | *pEXT22, Ptac::minC/minD, Spc^r^*  *pACYC, ftsZ^+^, AmP^r^*  *pDSW208, Ptrc::sulA, Amp^r^*  *pGB2 (repA^TS^), ftsZ^+^, Cam^r^*  *Plac::T25, Kan^r^*  *pQE80, Plac::6×his-slmA, Amp^r^*  *pUC18, with SBS12-SBS17 Kan^r^*  *pBAD18, Para::ftsZ, Amp^r^*  *pBAD18, Plac::6×his-ftsZ, Amp^r^*  *pEXT22, Ptac::slmA Spc^r^ (RBS: AGGAGG)*  *pEXT22, Ptac::slmA, Spc^r^*  *pQE80, Plac::zipA_185-328,_ Amp^r^*  *pKNT25, Plac::slmA-T25, Kan^r^*  *pUT18, Plac:;slmA-T18, Amp^r^*  *pE-SUMO,P_T7_::his-SUMO-SlmA, Amp*  *pUC18, Kan^r^*  *Plac::T18, Amp^r^*  *pKNT25, Plac::ftsZ-T25, Kan^r^*  *pUT18, Plac::ftsZ-T18, Amp^r^* | R100  pACYC  ColE1  pGB2  p15A  ColE1  ColE1  ColE1  ColE1  R100  R100  ColE1  p15A  ColE1  ColE1  ColE1  ColE1  p15A  ColE1 | [[15](#_ENREF_3)]  [[15](#_ENREF_2)]  [[15](#_ENREF_2)]  [[40](#_ENREF_3)]  This study  This study  Pichoff, S  This study  This study  This study  This study  This study  This study  This study  This study  This study  This study  This study  This study |
