# Supplementary material for: SlmA Antagonism of FtsZ Assembly Employs a Two-pronged Mechanism like MinCD
Source: PLoS Genet. 2014 Jul 31;10(7):e1004460. doi: 10.1371/journal.pgen.1004460 (PMC4117426; doi:10.1371/journal.pgen.1004460)
Supplement: Table S7 — List of SBS DNA molecules used in this study. (DOCX) [file pgen.1004460.s018.docx]

Table S7. List of SBS DNA molecules used in this study.

| Name of SBS DNA molecules | Sequence of SBS DNA molecules | Source |
| --- | --- | --- |
| SBS17-30mer | CAAAAGTAA**GTAAATGGTCAC**TAACGTTGA | [[17](#_ENREF_15)] |
| SBS17-20mer | GTAA**GTAAATGGTCAC**TAAC | This study |
| SBS-18mer | CCCAAT**GTGAGTGCTCAC** | [[20](#_ENREF_18)] |
| SBS-14mer | A**GTGAGTACTCAC**T | [[20](#_ENREF_18)] |
